# Efficacy and safety of hydroxyurea therapy on patients with *β*-thalassemia: a systematic review and meta-analysis

**DOI:** 10.3389/fmed.2024.1480831

**Published:** 2025-01-15

**Authors:** Tianmin Huang, Huixian Jiang, Ganling Tang, Jingyi Li, Xiaoman Huang, Zhenguang Huang, Hongliang Zhang

**Affiliations:** Department of Pharmacy, The First Affiliated Hospital of Guangxi Medical University, Nanning, China

**Keywords:** β-thalassemia, hydroxyurea, efficacy, safety, meta-analysis

## Abstract

**Objective:**

Our aim is to review the safety and efficacy of hydroxyurea (HU) on *β*-thalassemia patients.

**Methods:**

Studies that evaluated the safety and efficacy of HU on β-thalassemia patients were searched in Pub-Med, Cochrane Databases, Web of Science, China-Biology-Medicine, CNKI, Embase, VIP, and WanFang data. The proportions of response rate (RR) (50% fall in transfusion need in transfusion-dependent *β*-thalassemia patients, or 1 g/dL elevate in hemoglobin (Hb) levels in transfusion-independent *β*-thalassemia patients) and good RR (transfusion-free in transfusion-dependent *β*-thalassemia patients or 2 g/dL elevate in Hb levels in transfusion-independent β-thalassemia patients) were utilized to evaluate the effect size (ES). The secondary outcomes were the adverse events incidence rates of HU in *β*-thalassemia patients.

**Results:**

Two randomized controlled trials (RCTs) and 25 single-armed observational studies with typically 1,748 individuals were involved in our analysis. All 27 clinical trials were reported with fair quality. HU, in transfusion-dependent *β*-thalassemia patients, was related to a significant decrease in transfusion requirements [a pooled RR of 0.37 and a pooled good RR of 0.65 (95% CI, 0.53–0.76)]; in transfusion-independent *β*-thalassemia patients, it was correlated to an excellent raise in Hb levels [a pooled RR of 0.20 (95% CI, 0.08–0.35) and a pooled good RR of 0.53 (95% CI, 0.41–0.65)]. Neutropenia and leucopenia were the most prevalent adverse events in *β*-thalassemia patients treated with HU, while the incidence rates of other side effects were relatively lower.

**Conclusion:**

Our findings demonstrated that *β*-thalassemia patients tolerated and responded well to HU. Due to the control arms absence in the involved studies, more double-masked RCTs are essential for proving the safety and efficacy of HU in *β*-thalassemia patients.

## Introduction

Thalassemia is a prevalent global disease with a single-gene disorder (1). Thalassemia heterozygosity provides some immunity against malaria; however, a particularly high development of thalassemia in Central Asia, India, the Middle East, and Southern China is recognized. Meanwhile, *β*-thalassemia is widespread among people from these regions; those geographical areas are no longer limited as a result of migration to several places in the world (2, 3). Thalassemia syndromes include *α* and *β* gene disorders, resulting in α and β thalassemia, respectively, which are both inherited in an autosomal recessive manner (4). The *β*-thalassemia genotype is generally more severe than that of *α*-thalassemia. β-thalassemia comprises thalassemia minor due to single gene mutation and homozygous state in terms of double mutated genes and could be categorized by thalassemia major (*β*-TM) or intermedia (*β*-TI) according to clinical manifestations of patients. Furthermore, heterozygous genotypes, such as sickle cell *β*-thalassemia (S/β) and hemoglobin β-thalassemia (HbE/β), are observed. Currently, β-thalassemia is categorized into transfusion-dependent and transfusion-independent thalassemia (4).

Bone marrow and hematopoietic stem cell transplantation (SCT) are the rare medicinal ways for *β*-thalassemia nowadays due to the difficulty in identifying suitable donors and the relatively high cost. Moreover, gene therapy, an optimistic approach to cure thalassemia patients, could not be widely promoted and applied because its early-stage clinical trials have not been completed yet (5, 6). *β*-TM, severe β-TI, and severe HbE/β patients need a regular blood transfusion to maintain hemoglobin (Hb) and fetal hemoglobin (HbF) levels (6). However, significant risks such as acute life-threatening events (acute hemolytic reaction, bacterial infection, and anaphylaxis) are observed, and infections accompany chronic blood transfusions; meanwhile, iron remover may be required to avoid significant multi-system organ damage caused by iron overload (7).

Over the years, researchers have found that HbF inducer could reduce the anemia severity in *β*-thalassemia patients by elevating globin chain production and improving the excess chain imbalance. Several HbF inducers, such as erythropoietin, demethylating agents, 5-azacytidine, and short-chain fatty acids, have been studied in different combinations and individually to evaluate the clinical efficacy on *β*-thalassemia patients (8, 9). Hydroxyurea (HU), an impressively effective HbF inducer authorized by the U.S. Food and Drug Administration (FDA) in 1998, has been extensively used for sickle cell anemia patients. However, the use of HU in thalassemia was still restricted (10).

Many studies have shown that HU can significantly increase Hb and HbF levels in *β*-thalassemia patients, and no serious adverse events have been reported within a specific dose range (11, 12). Many meta-analyses described the HU treatment in lifelong transfusion-dependent (7, 13, 14), transfusion-independent (15, 16), and HbE/*β* (17) thalassemia patients. However, due to a single genotype of β-thalassemia, these studies lacked adequate power to identify the efficacy of HU for *β*-thalassemia patients with diverse genotypes or blood transfusion requirements. Furthermore, apart from one systematic review (18), which only included *β*-TM patients, no meta-analysis assessed the safety and efficacy of HU for β-thalassemia patients in the same article.

To estimate the safety and efficacy of HU on *β*-thalassemia patients from a relatively integrated viewpoint, we performed this meta-analysis, including clinical trials on the safety and efficacy of HU in β-thalassemia patients treatment with no limits to blood requirements or genotypes.

## Methods

### Data sources and searches

To assess the clinical safety and efficacy of HU on *β*-thalassemia patients of any age, we implemented a comprehensive systematic literature search. In this study, we searched Embase, Pub-Med, Web of Science, Cochrane Databases, China-Biology-Medicine, CNKI, VIP, WanFang data, and other databases using the following keywords: “thalassemia,” “*β*-thalassemia,” “thalassemia intermedia,” “thalassemia major,” “thalassemia minor,” “thalassemia syndrome,” “thalassemia trait,” “hydroxyurea” and “hydroxycarbamide” with a deadline of February 1^st^, 2022.

Additionally, manual searches were executed utilizing the reference lists of the original studies. Searches were unrestricted by publication type or date but restricted to human participants.

### Study selection

#### Eligibility criteria

Studies: Randomized clinical trials (RCTs) and observational studies (sample size >5 patients).

Publication language: English and Chinese.

Intervention: HU alone treatment.

Patients: *β*-thalassemia patients.

Our patients were separated into two groups dependent on their blood transfusion needs, and considering that the efficacy of HU treatment for thalassemia patients was evaluated by the reduction in blood transfusion need or increase in the Hb level.

For transfusion-dependent thalassemia patients, the blood transfusion frequency was more than eight times/year (7, 8, 19).

For transfusion-independent thalassemia patients, the blood transfusion frequency was less than four times/year, or they never had a blood transfusion history (20).

#### Primary outcomes

1. Transfusion-dependent *β*-thalassemia patients.

Response rate (RR): The lower blood transfusion frequency yearly ≥50%.

Good RR: Completely transfusion-free.

2. Transfusion-independent *β*-thalassemia patients.

RR: The elevated hemoglobin level ≥ 1 g/dL.

Good RR: The elevated hemoglobin level ≥ 2 g/dL.

#### Secondary outcomes

Adverse events in *β*-thalassemia patients.

#### Exclusion criteria

Patients who were not diagnosed with β-thalassemia.

Studies with a sample size of less than five patients.

Combination therapy with HU.

Case series.

Case reports.

### Data extraction

1. Transfusion-dependent thalassemia patients.

Author title, year of publication, country, disease, HU dose, design, age of first blood transfusion, mean age, response rate, and good response rate.

1. Transfusion-independent thalassemia patients.

Author title, year of publication, country, disease, HU dose, mean age, follow-up, study size, response rate, good response rate, and an increase of Hb level in responders.

1. Adverse events in *β*-thalassemia patients.

Myelosuppression, Hematologic effects (Neutropenia, Thrombocytopenia, Leucopenia), Neurologic, Dermatologic, and Gastrointestinal effects.

### Assessment of bias risk and evidence certainty assessment

Relying on the criteria outlined in the Cochrane Handbook for Systematic Reviews of Interventions (21), we recognized the bias risk for each RCT. These criteria included random sequence generation, completeness of the outcome data, allocation concealment, selective reporting bias, blinding of participants, and other sources of bias.

For non-randomized trials, an assessment of the bias risk was conducted via the Methodological Index For Non-Randomized Studies (MINORS) (22). Supplementary material S1 reveals that the principle had eight items for non-randomized studies without the control group and 12 items for non-randomized studies with the control group. These items were scored 0 (not reported), 1 (reported but inadequate), or 2 (reported and adequate). The ideal score was 24 for studies with a control group and 16 for studies without a control group.

Two review authors independently estimated the bias of each study, and any disagreements were resolved by communicating with the trial authors or by discussion with a third review author.

The online assessment conducted via the website (https://gdt.gradepro.org/app/) evaluates the certainty of the body of evidence concerning both primary and secondary outcomes.

### Statistical analysis

The RR of HU in *β*-thalassemia patients was the primary effect size (ES) of our meta-analysis, which decreased the blood transfusion requirement or increased their Hb levels. They were categorized into RR and good RR. The pooled estimate of the treatment effect for each outcome was calculated as a proportion (responders and good responders over the treated sample size with HU), along with their 95% confidence intervals (CIs), respectively, because the majority of the published trials had single armed designs without control arms.

The secondary ES was determined by the incidence rate of adverse events of HU in *β*-thalassemia patients, together with their 95% CIs.

The random-effect model, which considered within-study and between-study variation, was utilized and produced a more conservative analysis than the fixed-effect model in light of the expected heterogeneity within and between studies.

HU dose, Country, *β*-thalassemia genotype, quoting the Chi^2^ test and *p*-value, and the I^2^ statistic were the subgroup analyses intended to be used for examining each significant heterogeneity. Leave-one-out analysis be used to confirm stable results.

The publication was evaluated graphically by the Egger test and funnel plots, which quantified the plot asymmetry.

All these analyses were conducted via the “meta prop” and “metan” Stata command (Stata 17.0), and a random-effect model created for the utilization of proportions as ES and an alpha level of *p* < 0.05 was considered statistically significant.

## Results

### Study characteristics

**Figure 1:**
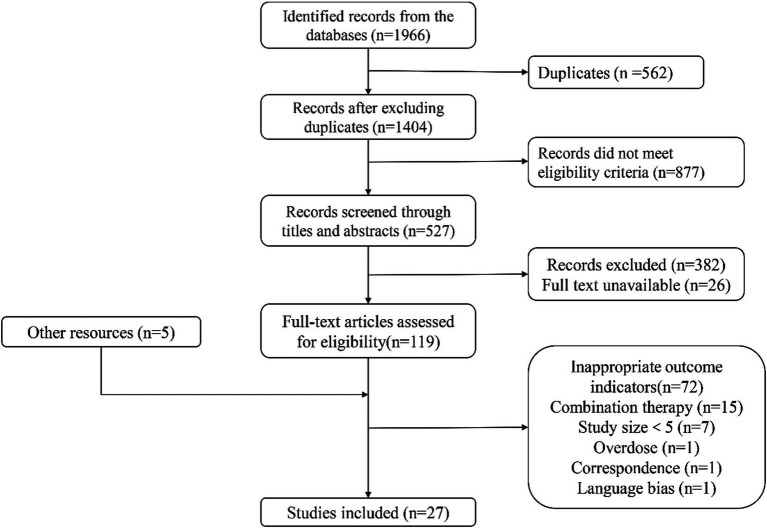
Study selection flow diagram.

The electronic search retrieved 1,966 citations, and 567 records were duplicates. Among the remaining 1,404 articles, 877 records were excluded for specific exclusion rules. For further reading, 527 articles were listed below; 382 records were excluded because their research objectives or methods did not meet expectations; 22 records could not be obtained, or formal studies had not been published yet; accordingly, only 123 articles could be read in full text. In addition, five articles for further reading were manually retrieved from other channels. Among these 128 articles, 101 were excluded according to inclusion criteria and specific regulations. Finally, 27 clinical trials were included in this study (Figure 1).

Our analysis included a total of 27 studies (11, 23–48), involving two RCTs (25, 30) and 25 single-armed studies (11, 23, 24, 26–29, 31–48). Nineteen studies (23, 24, 26, 30, 31, 33–43, 45, 47, 48) involving 1,352 transfusion-dependent patients, 15 studies (23, 24, 26, 30, 31, 33, 35, 36, 38–42, 47, 48) with a total of 1,054 patients reported RR, 17 studies (23, 26, 30, 31, 33–37, 39–43, 45, 47, 48) with a total of 1,201 patients reported good RR; 11 studies (11, 23, 25, 27–29, 31, 32, 40, 44, 46) involving 396 transfusion-independent patients, 11 studies (11, 23, 25, 27–29, 31, 32, 40, 44, 46) with totally 396 patients reported RR, six studies (27–29, 31, 32, 40) with totally 254 patients reported good RR Tables 1, 2 manifests the features of the included studies.

**Table 1:** Characteristics of transfusion-dependent β-thalassemia patients.

Study-year	Country	Design	Genotype	HU dose(mg/kg/d)	Frequency of blood transfusion	Mean age (y) ± SD (range)	Follow-up(m)	No. of participants	Response	Good response
Ansari 2011	Pakistan	Single armed observational	TM	16	>8/y	7.3	24	119	90	33
Biswas 2019	India	Single armed observational	HbE/β	10–20	Regular	15.6 ± 11.5	28	110	42	NA
Bordbar 2014	India	Single armed observational	TM	10.5	Every 2–4 weeks	20.27 ± 8.3 (2–50)	3–14	95	31	6
Elalfy 2013	Egypt	RCT	TI	25	Monthly	9.1 ± 3.7 (5–17.5)	12	40	25	6
El-Beshlawy 2014	Egypt	Single armed observational	TI	10–29	Frequently	4.11 ± 1.68 (1–8)	4–96	25	23	11
Hashemi 2009	Iran	Single armed observational	TM, TI	10–15	Monthly	16.5 (3–40)	24	91	38	17
Huang 2016	China	Single armed observational	TI	10–30	NA	25. 59 (5–67)	12	12	NA	10
Iqbal 2018	Pakistan	Single armed observational	βT	16–20	>8/y	9.93 ± 6.68	6–12	70	48	25
Italia 2009	India	Single armed observational	TM	15–20	NA	12–28	20–24	41	13	NA
Italia 2010	India	Single armed observational	HbE/β	15–20	12-15/y	8–34	20	11	NA	4
Italia 2013	India	Single armed observational	TM, HbE/β	10–15	12-15/y	5–30	24	11	7	2
Karimi 2005	Iran	Single armed observational	TI	8–12	12-48/y	13.5 (4–35)	72	120	106	83
Karimi 2012	Iran	Single armed observational	βT	8–15	NA	20.6 ± 7.4 (4–45)	156	126	95	51
Kattamis 2004	Greek	Single armed observational	S/β	15–30	Regular	17 ± 6.2 (8.8–27.1)	24	8	2	0
Koren 2008	Israel	Single armed observational	TM, TI	10.9 ± 3	30-71(cc/kg/year)	22.6 ± 7 (9–34)	48	16	14	14
Kosaryan 2009	Iran	Single armed observational	TM	15.5 ± 6.4	NA	17.5	89.5	248	NA	111
Singer 2005	United States, Canada, United Kingdom	Single armed observational	Hb E/β	18–20	Regular	13.2 (1.2–35)	36	27	NA	12
Yavarian 2004	Iran	Single armed observational	TM	10–15	Monthly	17.1 ± 5.2 (8–31)	60	133	112	81
Zamani 2009	Iran	Single armed observational	TM	8–15	3-4 weeks	18.38 (10–40)	60	49	44	12

**Table 2:** Characteristics of transfusion-independent β-thalassemia patients.

Study-year	Country	Design	Genotype	HU dose (mg/kg/d)	Mean age (y) ± SD (range)	Follow-up (m)	No. of participants	Response	Good response	Increase of Hb level in responders (g/dl)
Ansari 2011	Pakistan	Single armed observational	TI	16	5.4	24	27	12	NA	NA
Bohara 2014	India	RCT	Hb E/β	10	16.68 ± 6.05	6	32	18	NA	NA
Bohara 2014	India	RCT	Hb E/β	20	16.17 ± 5.75	6	29	5	NA	NA
De Paula 2003	Brazil	Single armed observational	TI	10–20	16–68	6–96	7	3	1	1.7
Dixit 2005	India	Single armed observational	TI	10–30	10 (4–50)	4–36	37	26	17	2.1
Ehsani 2009	Iran	Single armed observational	TI	20	10.7 ± 5.0 (5–19)	6	16	11	4	2.1
El-Beshlawy 2014	Egypt	Single armed observational	TI	10–29	4.11 ± 1.68 (1–8)	4–96	75	56	22	1.5
Fucharoen 1996	Thailand	Single armed observational	Hb E/β	10–20	34 (18–55)	5	13	3	0	1.6
Karimi 2012	Iran	Single armed observational	TI	8–15	20.6 ± 7.4 (4–45)	96–156	106	56	12	1.8
Panigrahi 2005	India	Single armed observational	TI	10–20	5–32	12	15	8	NA	2.4
Rigano 2010	Italy	Single armed observational	TI	5–30	37 (18–59)	35–180	24	17	NA	1.7
Singer 2008	United States, Canada, United Kingdom	Single armed observational	Hb E/β	18–20	13.7 ± 6 (3–27)	10.2	15	8	NA	1.5

From these studies, we extracted data on adverse events after treatment with HU on *β* -thalassemia. (Table 3). Sixteen studies (23–27, 30–32, 34, 36, 38, 40–43, 48) reported the incidence of adverse events. Among them, four studies (23, 30, 34, 41) reported myelosuppression, 12 studies reported hematological side effects (seven studies (25–27, 31, 36, 38, 42) reported neutropenia, three studies (24, 25, 48) reported thrombocytopenia, three studies (25, 32, 43) reported leucopenia), one study (40) reported neurological effects, one study (40) reported dermatologic effects and six studies (23, 25, 34, 40, 43, 48) reported gastrointestinal effects.

**Table 3:** Adverse events in β-thalassemia patients.

Study	No. of participants	Myelosuppression	Hematologic effects			Neurologic effects	Dermatologic effects	Gastrointestinal effects
			Neutropenia	Thrombocytopenia	Leucopenia			
Ansari 2011	146	4	–	–	–	–	–	2
Biswas 2019	110	–	–	32		–	–	–
Bohara 2014	32	–	1	3	3	–	–	3
Bohara 2014	29	–	9	10	18	–	–	9
Bordbar 2014	97	–	16	–	–	–	–	–
De Paula 2003	11	–	1	–	–	–	–	–
El-Beshlawy 2014	100	–	2	–	–	–	–	–
Elalfy 2013	40	1	–	–	–	–	–	–
Fucharoen 1996	13	–	–	–	3	–	–	–
Huang 2016	14	3	–	–	–	–	–	1
Italia 2009	79	–	12	–	–	–	–	–
Italia 2013	16	–	2	–	–	–	–	–
Karimi 2012	232	–	–	–	–	23	28	6
Kattamis 2004	8	2	–	–	–	–	–	–
Koren 2008	18	–	3	–	–	–	–	–
Kosaryan 2009	248	–	–	–	19	–	–	3
Zamani 2009	49	–	–	1	–	–	–	8

### Assessment of bias risk and evidence certainty

Supplementary material S2 shows the bias risk assessment by the Cochrane Handbook for RCTs, with no significant bias.

Supplementary material S3 presents the assessment of the bias risk for non-randomized trials. According to the MINORS, all the non-randomized trials were rated above eight points, with no obvious overall bias risk implicated for any included study.

Except for the study by De Paula et al. (27), which has low certainty of evidence due to its small sample size, most other studies have moderate or high certainty of GRADE evidence.

### Primary outcomes

#### Transfusion-dependent *β*-thalassemia patients

**Figure 2:**
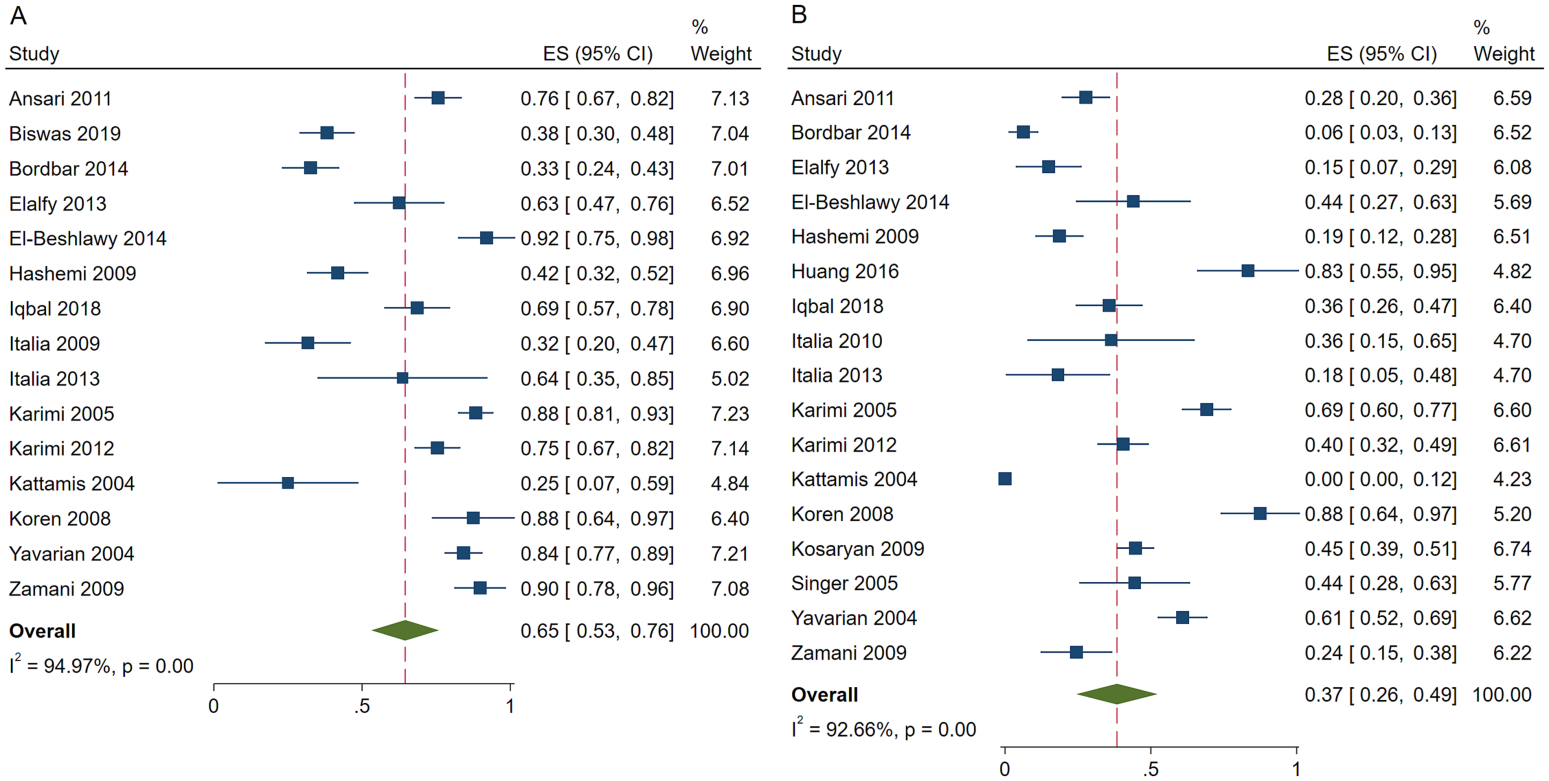
Forest plots of HU responses in transfusion-dependent β-thalassemia patients. **(A)** Forest plot of the response rate of HU in transfusion-dependent β-thalassemia patients. **(B)** Forest plot of the good response rate of HU in transfusion-dependent β-thalassemia patients.

The pooled ES of RR was 0.65 [95% CI, 0.53–0.76], I^2^ = 94.97%, with a high degree of heterogeneity; consequently, the random effect was used (Figure 2). Given the significant heterogeneity observed in the results, a subgroup analysis was conducted. Patients in Israel had the highest ES of 0.88 [95% CI, 0.64–0.97] relying on the outcomes of the subgroup analysis, while those receiving HU doses below 15 mg/kg/d had the highest ES of 0.71 [95% CI, 0.55–0.87] (Table 4). Our leave-one-out analysis confirmed stable results.

**Table 4:** Subgroup analysis of the response rate of HU in transfusion-dependent β-thalassemia patients.

Variable	No. of studies	No. of participants	No. of response	ES	95%CI	*I*^2^/%	*p*
Country							<0.01
Pakistan	2	189	138	0.73	0.67–0.80	–	–
India	4	257	93	0.37	0.29–0.44	36.05	0.20
Egypt	2	65	48	0.82	0.73–0.91	–	–
Iran	5	519	395	0.76	0.62–0.90	94.37	<0.01
Greek	1	8	2	0.25	0.07–0.59	–	–
Isreal	1	16	14	0.88	0.64–0.97	–	–
Genotype							<0.01
TM	6	528	328	0.60	0.39–0.80	96.88	<0.01
TI	3	185	154	0.82	0.69–0.96	–	–
HbE/β	1	110	42	0.38	0.30–0.48	–	–
HbE/β, TM	1	11	7	0.64	0.35–0.85	–	–
TM, TI	1	16	14	0.88	0.64–0.97	–	–
S/β	1	8	2	0.25	0.07–0.59	–	–
βT	2	196	143	0.73	0.67–0.79	–	–
HU dose							0.26
≤ 15 mg/kg/d	7	630	440	0.71	0.55–0.87	96.29	<0.01
>15 mg/kg/d	8	424	250	0.58	0.42–0.75	92.49	<0.01

The pooled ES of good RR in transfusion-dependent *β*-thalassemia patients was 0.37 [95% CI, 0.26–0.49], I^2^ = 92.66%, with a high heterogeneity degree; therefore, the random effect was used (Figure 2). The highest ES was recorded by patients in Israel of 0.88 [95% CI, 0.64–0.97], and the highest ES was recorded by those who received HU doses below 15 mg/kg/d of 0.46 [95% CI, 0.26–0.67] (Table 5). Our leave-one-out analysis confirmed stable results.

**Table 5:** Subgroup analysis of the good response rate of HU in transfusion-dependent β-thalassemia patients.

Variable	No. of studies	No. of participants	No. of response	ES	95%CI	*I*^2^/%	*P*
Country							<0.01
Pakistan	2	189	58	0.31	0.24–0.37	–	–
India	3	117	12	0.16	0.01–0.40	–	–
Egypt	2	65	17	0.25	0.15–0.36	–	–
Iran	6	767	355	0.43	0.29–0.58	93.87	<0.01
Greek	1	8	0	0.00	0.00–0.32	–	–
Isreal	1	16	14	0.88	0.64–0.97	–	–
China	1	12	10	0.83	0.55–0.95	–	–
United States, Canada, United Kingdom	1	27	12	0.44	0.28–0.63	–	–
Genotype							<0.01
TM	6	735	260	0.29	0.14–0.47	95.68	<0.01
TI	4	197	110	0.52	0.21–0.82	93.29	<0.01
HbE/β	2	38	16	0.42	0.26–0.59	–	–
HbE/β, TM	1	11	2	0.18	0.05–0.48	–	
TM, TI	1	16	14	0.88	0.64–0.97	–	–
S/β	1	8	0	0.00	0.00–0.32	–	–
βT	2	196	76	0.39	0.32–0.46	–	–
HU dose							0.19
≤ 15 mg/kg/d	8	642	274	0.46	0.26–0.67	96.19	<0.01
>15 mg/kg/d	9	559	204	0.31	0.22–0.40	74.22	<0.01

HU was more effective for *β*-TI patients (*p* < 0.01) and had no significant variation in the efficacy of β-thalassemia patients with different HU dosages (*p* = 0.26, *p* = 0.19).

#### Transfusion-independent *β*-thalassemia patients

**Figure 3:**
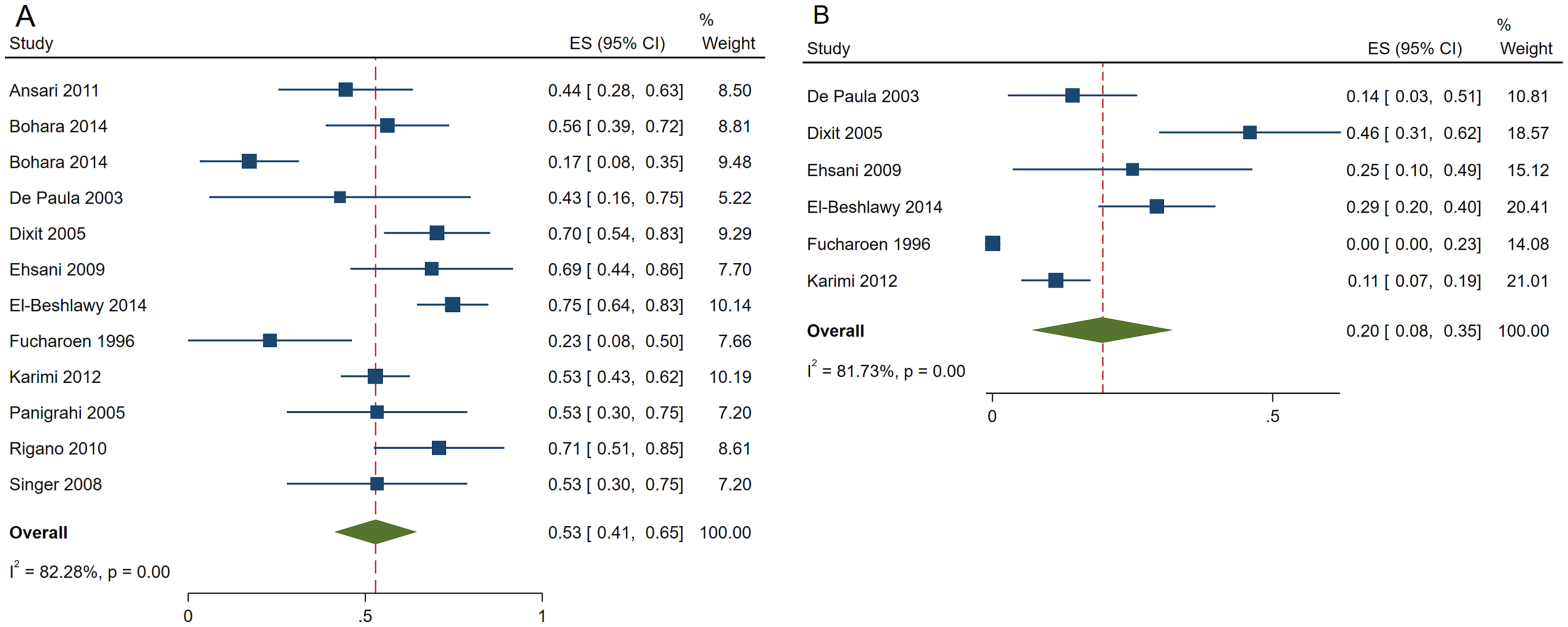
Forest plots of HU responses in transfusion-independent β-thalassemia patients. **(A)** Forest plot of the response rate of HU in transfusion-independent β-thalassemia patients. **(B)** Forest plot of the good response rate of HU in transfusion-independent β-thalassemia patients.

The pooled ES of RR was 0.53[95% CI, 0.41–0.65], *I*^2^ = 82.28%, with a high heterogeneity degree; consequently, the random effect was used (Figure 3). Patients in Egypt had the highest ES of 0.75 [95% CI, 0.64–0.83], *β*-TI patients had the highest ES, which was 0.62 [95% CI, 0.53–0.71], and patients received HU doses below 15 mg/kg/d had the highest ES, which was 0.54 [95% CI, 0.45–0.62] (Table 6). Our leave-one-out analysis confirmed stable results.

**Table 6:** Subgroup analysis of the response of HU in transfusion-independent β-thalassemia patients.

Variable	No. of studies	No. of participants	No. of response	ES	95%CI	*I*^2^/%	*P*
Country							<0.01
Pakistan	1	27	12	0.44	0.28–0.63	–	–
India	4	113	57	0.49	0.23–0.75	89.63	<0.01
Brazil	1	7	3	0.43	0.16–0.75	–	–
Iran	2	122	67	0.55	0.46–0.64	–	–
Egypt	1	75	56	0.75	0.64–0.83	–	–
Thailan	1	13	3	0.23	0.08–0.50	–	–
Italy	1	24	17	0.71	0.51–0.85	–	–
United States, Canada, United Kingdom	1	15	8	0.53	0.30–0.75	–	–
Genotype							0.04
TI	8	307	189	0.62	0.53–0.71	59.24	0.02
HbE/β	4	89	34	0.37	0.15–0.58	80.28	<0.01
HU dose							0.89
≤ 15 mg/kg/d	2	138	74	0.54	0.45–0.62	–	–
>15 mg/kg/d	10	258	149	0.52	0.37–0.68	85.41	<0.01

The pooled ES of the good RR in transfusion-independent *β*-thalassemia patients was 0.20 [95% CI, 0.08–0.35], *I*^2^ = 81.73% with a high heterogeneity degree; subsequently, the random effect was used (Figure 3). *β*-TI patients had the highest ES of 0.25 [95% CI, 0.11–0.41] and patients receiving HU above 15 mg/kg/d had the highest ES of 0.22 [95% CI, 0.08–0.40], depending on the findings of subgroup analysis. Patients from India had the highest ES of 0.46 [95% CI, 0.31–0.62] (Table 7). Our leave-one-out analysis confirmed stable results.

**Table 7:** Subgroup analysis of the good response of HU in transfusion-independent β-thalassemia patients.

Variable	No. of studies	No. of participants	No. of response	ES	95%CI	*I*^2^/%	*P*
Country							<0.01
Brazil	1	7	1	0.14	0.03–0.51	–	–
India	1	37	17	0.46	0.31–0.62	–	–
Iran	2	122	16	0.12	0.07–0.19	–	–
Egypt	1	75	22	0.29	0.20–0.40	–	–
Thailand	1	13	0	0.00	0.00–0.23	–	–
Genotype							0.01
TI	5	241	56	0.25	0.11–0.41	80.69	<0.01
HbE/β	1	13	0	0.00	0.00–0.23	–	–
HU dose							0.11
≤ 15 mg/kg/d	1	106	12	0.11	0.07–0.19	–	–
>15 mg/kg/d	5	148	44	0.22	0.08–0.40	74.02	<0.01

β-thalassemia patients from Egypt [ES = 0.75 (95% CI, 0.64–0.83)] and India [ES = 0.46 (95% CI, 0.31–0.62)] had better responses to HU (*p* < 0.01).

HU was more effective for β-TI patients (*p* = 0.04, *p* = 0.01) and had no significant variance in the efficacy of β-thalassemia patients with different HU dosages (*p* = 0.89, *p* = 0.11).

### Secondary outcomes

#### Adverse events

Four studies reported myelosuppression, with a combined effect value of 0.04 [95% CI, −0.01 – 0.08]; 12 studies reported hematological side effects; among them, seven studies reported neutropenia with a combined effect value of 0.12 [95% CI, 0.05–0.18], three studies announced thrombocytopenia with combined effect value of 0.18 [95% CI, 0.02–0.34], three studies announced leucopenia with a combined effect value of 0.24 [95% CI, 0.05–0.43], one study reported neurological side effects with a combined effect value of 0.10 [95% CI, 0.07–0.14], one study reported dermatologic effects with a combined effect value of 0.12 [95% CI, 0.08–0.17], and six studies reported gastrointestinal side effects with a combined effect value of 0.04 [95% CI, 0.01–0.07] (Table 8).

**Table 8:** Pooled proportion of the adverse events of HU in β-thalassemia patients.

Adverse events	No. of trials	No. of participants	No. of events	ES	95%CI	*I*^2^/%	*P*
Myelosuppression	4	208	10	0.04	−0.01-0.08	–	–
Neutropenia	7	382	46	0.12	0.05–0.19	–	–
Thrombocytopenia	3	220	46	0.17	−0.02-0.36	–	–
Leucopenia	3	322	43	0.21	0.01–0.41	–	–
Neurologic effects	1	232	23	0.10	0.07–0.14	–	–
Dermatologic effects	1	232	28	0.12	0.08–0.17	–	–
Gastrointestinal effects	6	750	32	0.04	0.01–0.07	–	–

Although neutropenia and gastrointestinal effects were reported in most studies, the overall incidence of these side effects was lower compared to other treatment-related adverse events. Thrombocytopenia and leucopenia were reported in some studies with relatively high incidences of 0.17 [95% CI, −0.02 – 0.36] and 0.21 [95% CI, 0.01–0.41], respectively. One study reported neurological and dermatologic effects with higher incidence rates of 0.10 [95% CI, 0.07–0.14] and 0.12 [95% CI, 0.08–0.17], respectively. According to study of Iran region (40, 49), the presence of adverse effects in patients significantly increased by increasing age (*p* < 0.001) and splenectomy (*p* < 0.05), but it had no significant relationship with sex, HU dose, ethnic, or duration of treatment (*p* > 0.05).

#### Assessment of reporting bias

**Figure 4:**
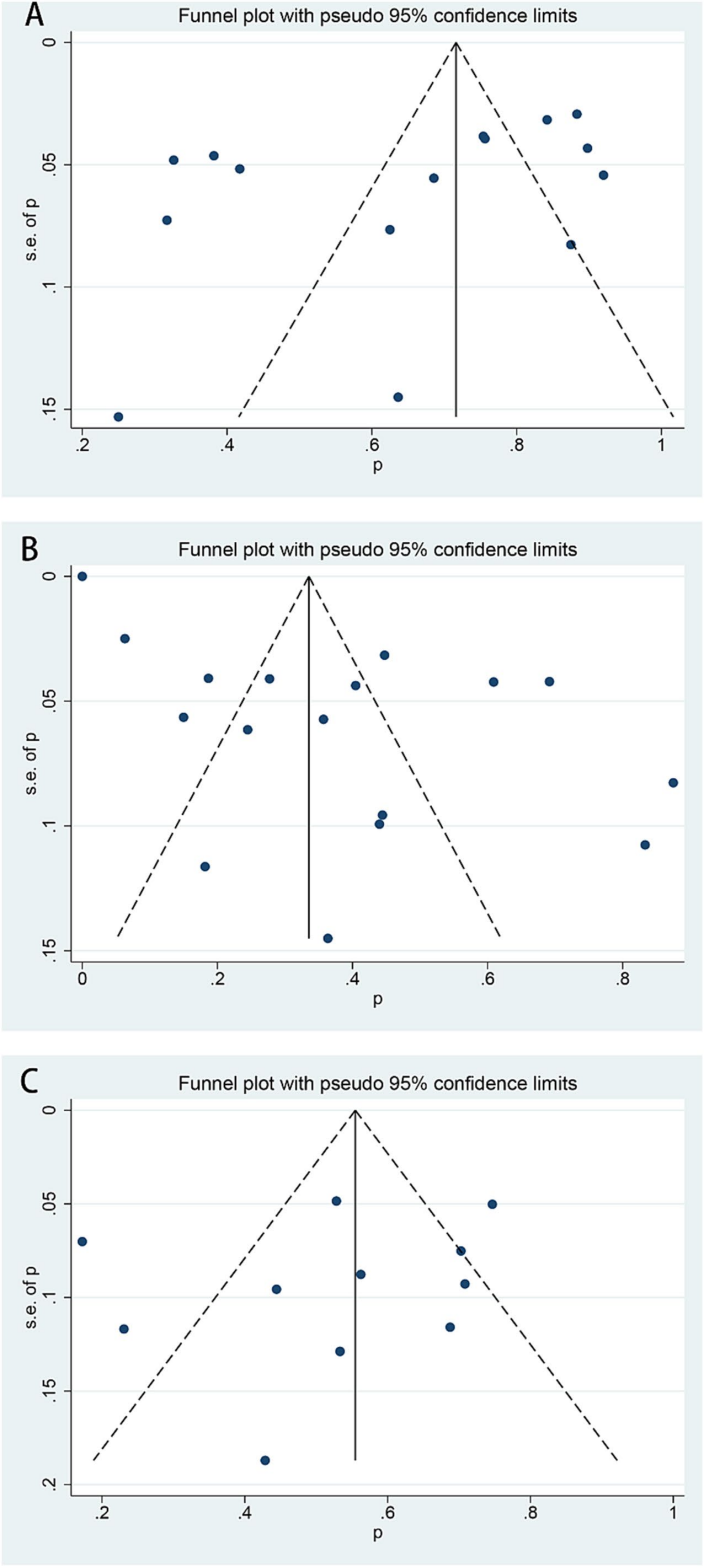
Funnel plots of HU responses in β-thalassemia patients. **(A)** A response rate of transfusion-dependent β-thalassemia patients, Eggar test *p* = 0.109. **(B)** The good response rate of transfusion-dependent β-thalassemia patients, Egger test *p* = 0.155. **(C)** A response rate of transfusion-independent β-thalassemia patients, Egger test *p* = 0.481.

According to the results of funnel plots, as well as the Egger test (Figure 4), no reporting bias was observed in this study (Figure 4).

## Discussion

This study evaluated the safety and efficacy of HU in *β*-thalassemia patients for 1,352 patients who were blood transfusion-dependent and 396 patients who were blood transfusion-independent. The RR of HU in transfusion-dependent patients was 0.65 [95% CI, 0.53–0.76], and the good RR was 0.37 [95% CI, 0.26–0.49]; in transfusion-independent patients, the RR of HU was 0.53 [95% CI, 0.41–0.65] and the good RR was 0.20 [95% CI, 0.08–0.35]. In general, *β*-thalassemia patients could benefit from HU.

In previous studies, Algiraigri et al., published a series of meta-analyses about different genotypes of *β*-thalassemia patients treated with HU, including lifetime transfusion-dependent thalassemia (7), transfusion-independent thalassemia (15) and HbE/β thalassemia (17), furthermore using an analysis of the overall effect of value extracting the figures of single arms. In line with their findings, we also endorse the utilization of HU. In addition, two published meta-analyses, including RCTs, were found in the *Cochrane Database of Systematic Reviews.* One of them just reported transfusion-dependent β-thalassemia patients (14) and did not enroll any literature; another one, dependent on transfusion-independent β-thalassemia patients (16), enrolled one literature (25), which was included in our study.

Blood transfusion is a recommended therapy for *β*-thalassemia patients, a hereditary disease that has long afflicted the world (50); however, numerous problems like iron excesses accompany this method. Subsequently, iron agents are required after blood transfusion. Other treatments, such as SCT and gene therapy, have been approved to be significantly effective; nevertheless, they were difficult to implement (6) due to the economic and operational constraints of these therapies make them unsuitable for patients from ordinary families or developing countries, leading to a need for more studies on the most convenient and cost-effective treatment (51). Currently, HbF inducer is an increasingly powerful drug that elevates HbF levels by enhancing the severity of *β*-thalassemia patients and reducing the imbalance of *α*/*β* chain. One of them is HU, a globin synthesis inducer, which leads to stress erythrocyte production through its cytotoxicity, resulting in increased HbF levels (8).

The FDA authorized HU as a key drug for sickle cell disease treatment; however, the HU use in thalassemia patients has not been confirmed. Numerous studies have shown that HU can effectively lower the blood transfusion frequency in thalassemia patients (23, 39, 40, 43), raise the level of Hb (11, 28, 29, 31), improve the HbF level (32, 48, 52), and improve their quality of life (53, 54). Meanwhile, The data collected were RR and good RR in *β*-thalassemia patients who received HU. The combined RRs were above 50% for both transfusion-dependent and transfusion-independent *β*-thalassemia patients, which showed significant curative impacts of HU on β-thalassemia patients.

Consistent with the description in the introduction, most of the people included in this study came from Southest Asia and West Asia. Among them, 6 studies of Iranian (total 19 studies) reported transfusion-dependent patients, while 4 studies of Indian (total 11 studies) reports transfusion-independent patients. Many types of *β*-thalassemia patients were included in our study. Herein, HU significantly reduced or ceased blood transfusion of transfusion-dependent *β*-TM patients and significantly improved the Hb level of transfusion-independent β-TI patients. Moreover, in comparison to HU dose above 15 mg/kg/d, the efficacy was better when using HU less than 15 mg/kg/d in transfusion-dependent β-thalassemia patients. However, no statistically significant variation was identified. While HU’s effectiveness in treating β-thalassemia differs by country and genotype (*p* < 0.05), it can be applied to other populations or settings with regular blood monitoring due to its efficacy and safety. A 13-year experience in Iran suggested that HU at a dose of 8–15 mg/kg/day was effective in decreasing or effecting cessation of the need for regular blood transfusion, as well as in increasing Hb levels in *β*-thalassemia patients, without any major side effects (40). Another study found that the response of HU treatment is best in patients having both XmnI polymorphism and *α*-deletion (24). Several HbF inducers have also been successfully tried in thalassemia syndromes besides HU. These include 5-Azacytidine, Butyrates, Erythropoietin etc. (4) All these agents have shown promising results in thalassemia, but to a lesser extent in thalassemia major. Additionally, combining hydroxyurea with recombinant human erythropoietin (rHuEPO) shows promise by significantly boosting hemoglobin levels and reducing blood transfusion needs in *β*-thalassemia patients, while maintaining good safety (30). Based on what was above, long-term HU efficacy, optimal dosing for different genotypes, and combined therapies could be crucial points for researchers to dig out and clear off in future.

Parts of the articles retrieved showed that HU was effective for *β*-thalassemia patients (55–57); however, the full texts were unavailable, resulting in an exclusion for incomplete data. In addition, after the search deadline, we found a high-quality placebo-controlled RCT (58) of 10-20 mg/kg/d oral hydroxyurea for transfusion-dependent β-thalassaemia, which made a comparison of amount of blood volume, the percentage of fetal hemoglobin percentage and erythropoietic stress as measured by soluble transferrin receptor concentration between two groups, indicating that HU was effective in increasing HbF levels and decreasing blood transfusions in β-thalassemia patients, but ultimately excluded it due to inconsistent inclusion criteria with our study.

To evaluate the safety of β-thalassemia patients who received HU, we focused on the incidence of bone marrow suppression and hematological, neurological, dermatologic, and gastrointestinal side effects. Myelosuppression is a relatively common bone marrow toxic effect of HU, while previous studies of HU used for thalassemia reported little myelosuppression in some studies (49, 59). Among the articles reporting myelosuppression in this study, myelosuppression in patients was reversible (41); one patient recovered after reducing HU dose (30), and some patients with myelosuppression entirely recovered after cessation of drug therapy (23, 34). Leucopenia was the most frequent adverse event in hematology effects, followed by neutropenia (Table 8). Neurologic and dermatologic effects adverse effects were less reported, and gastrointestinal effects (mainly nausea, vomiting, and diarrhea) were more reported. Kattami et al. (60) reported that dermatologic effects like pigmentation, alopecia, and macular papules were most common, and gastrointestinal side effects such as tarry stools and diarrhea were second most common in included patients. Simultaneously, the product specification (61) for HU lists common adverse reactions affecting the hematologic, gastrointestinal symptoms, nervous system, and skin. Additionally, in a study of 299 sickle cell anemia patients (62), the most frequent side effects were hematologic, including neutropenia and reduced reticulocyte and platelet levels. This might be due to HU’s inhibition of DNA synthesis and cell growth, impacting bone marrow’s hematopoietic function, directly stimulating gastrointestinal epithelial cells, and triggering oxidative stress and inflammation, leading to adverse reactions. Although start dose of HU was 10 mg/kg per day and increased by 5 mg/kg per day every 4–6 weeks until toxicity or according to clinical response, the study reported no significant correlation between the HU dosage and the occurrence of adverse effects (49). It is worth mentioning that our study demonstrated that *β*-thalassemia patients who received HU had a low incidence of adverse events, which were mild and recoverable. If it was possible, we could compare response rates between dependent and independent β-thalassemia patients, or both with individuals without β-thalassemia in same dose of hydroxyurea in future studies.

The limitations of our study includes that firstly, most of the articles about HU in the *β*-thalassemia treatment were single-armed and retrospective observational studies, which present clear limitations in research design, such as the absence of parallel control groups and limited comparability. Secondly, the use of external controls may introduce confounding variables that could compromise the integrity of the evaluation. Thirdly, the subgroups were categorized by HU dose, country, and β-thalassemia genotype, with complete data on these characteristics. Sensitivity analysis and bias assessment support the study’s primary outcomes. However, the unavailability of some primary outcomes in the included studies may impact the accuracy and reliability of the analysis. Multicenter RCTs (compared with placebo or other available treatments, such as iron chelation and blood transfusion) with large sample size are needed to evaluate the safety and efficacy of *β*-thalassemia patients treated with HU (14).

Although the outcome indicators did not clearly and directly verify the purpose of our study, and the quality of included studies was flawed, this study collected more studies as comprehensively as possible, included a wider range of population, and used subgroup analysis to make comparison between groups, explaining the drug use characteristics of different groups of population, and providing some reference for future research. Existing studies have shown that HU works well; moreover, it can be a key drug in the *β*-thalassemia treatment, which requires more economic-effect evaluations to evaluate the cost savings of HU in transfusion, management of iron-chelation-toxic-related diseases and equipment (18).

## Conclusion

β-thalassemia patients can benefit from HU therapy because it may raise Hb levels in transfusion-independent patients and lower the blood transfusion needs in transfusion-dependent patients. Meanwhile, neutropenia and leucopenia were the prevalent adverse events in β-thalassemia patients treated with HU; the incidence rates of other side effects were relatively lower, according to our study. It can be concluded that HU is safe and well-tolerated in β-thalassemia patients.

## Data Availability

The original contributions presented in the study are included in the article/Supplementary material, further inquiries can be directed to the corresponding author/s.
