# Supplementary material for: Efficacy and safety of hydroxyurea therapy on patients with β-thalassemia: a systematic review and meta-analysis
Source: Front Med (Lausanne). 2025 Jan 15;11:1480831. doi: 10.3389/fmed.2024.1480831 (PMC11774989; doi:10.3389/fmed.2024.1480831)
Supplement: Supplementary file 1 [file Data_Sheet_1.PDF]

Supplementary 1. Methodological items for non-randomised studies

**Methodological items for non-randomised studies**	Score†
1. **A clearly stated aim:** the question addressed should be precise and relevant in the light of available literature<br>2. **Inclusion of consecutive patients:** all patients potentially fit for inclusion (satisfying the criteria for inclusion) have been included in the study during the study period (no exclusion or details about the reasons for exclusion)<br>3. **Prospective collection of data:** data were collected according to a protocol established before the beginning of the study<br>4. **Endpoints appropriate to the aim of the study:** unambiguous explanation of the criteria used to evaluate the main outcome<br>which should be in accordance with the question addressed by the study. Also, the endpoints should be assessed on an intention-to-treat basis.<br>5. **Unbiased assessment of the study endpoint:** blind evaluation of objective endpoints and double-blind evaluation of subjective<br>endpoints. Otherwise the reasons for not blinding should be stated<br>6. **Follow-up period appropriate to the aim of the study:** the follow-up should be sufficiently long to allow the assessment of<br>the main endpoint and possible adverse events<br>7. **Loss to follow up less than 5%:** all patients should be included in the follow up. Otherwise, the proportion lost to follow up<br>should not exceed the proportion experiencing the major endpoint<br>8. **Prospective calculation of the study size:** information of the size of detectable difference of interest with a calculation of 95％ confidence interval, according to the expected incidence of the outcome event, and information about the level for statistical significance and estimates of power when comparing the outcomes<br><br>*Additional criteria in the case of comparative study*<br>9. **An adequate control group:** having a gold standard diagnostic test or therapeutic intervention recognized as the optimal intervention according to the available published data<br>10. **Contemporary groups:** control and studied group should be managed during the same time period (no historical comparison)<br>11. **Baseline equivalence of groups:** the groups should be similar regarding the criteria other than the studied endpoints. Absence<br>of confounding factors that could bias the interpretation of the results<br>12. **Adequate statistical analyses:** whether the statistics were in accordance with the type of study with calculation of confidence<br>intervals or relative risk	
†The items are scored 0 (not reported), 1 (reported but inadequate) or 2 (reported and adequate). The global ideal score being 16 for non-comparative studies and 24 for comparative studies.	

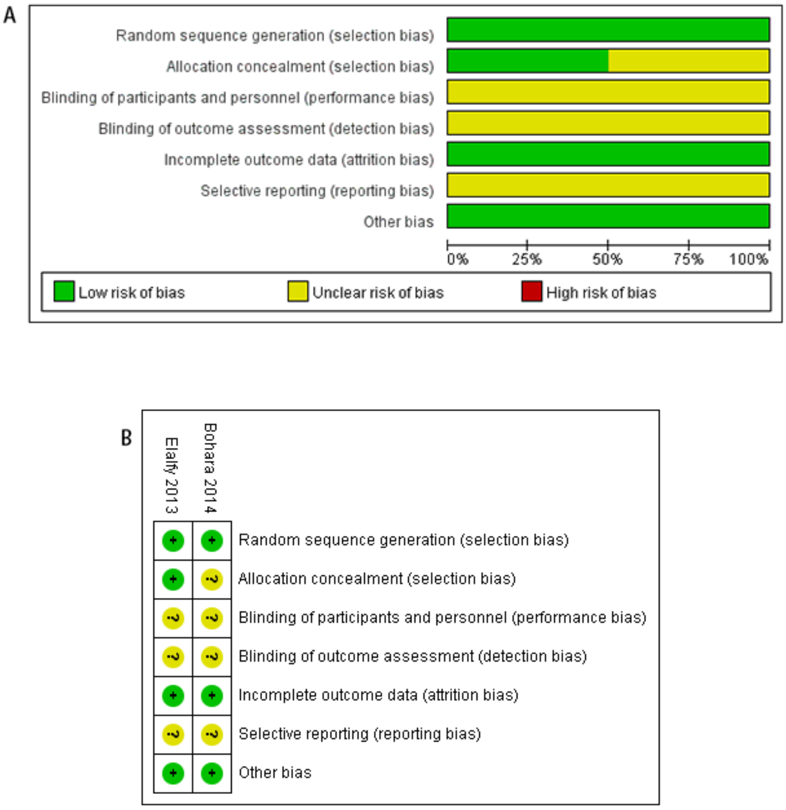

A: Risk of bias graph

B: Risk of bias summary

Supplementary 2: Risk of bias for randomised controlled trials

Supplementary 3. Risk of bias for non-randomised trials

Study/year	Ansari 2011	Biswas 2018	Bordbar 2014	De Paula 2003	Dixit 2005	Ehsari 2009	El-Beshla 2014	Fucharoen 1996	Huang 2016	Hashemi 2009	Iqbal 2018	Italia 2009	Italia 2010
A clearly stated aim	2	2	2	2	2	2	2	2	2	2	2	2	2
Inclusion of consecutive patients	2	2	2	2	2	2	2	0	1	2	2	2	2
Prospective collection of data	2	2	2	2	2	2	2	2	2	2	2	2	2
Endpoints appropriate to the aim of the study	2	2	2	2	2	2	2	1	1	2	1	2	2
Unbiased assessment of the study endpoint	0	0	0	0	0	0	0	0	0	0	1	0	0
Follow-up period appropriate to the aim of the study	2	2	2	2	2	1	2	1	2	2	2	2	2
Loss to follow up less than 5%	2	2	2	2	2	2	2	2	2	2	2	2	2
Prospective calculation of the study size	0	0	0	0	0	0	0	0	0	0	0	0	0
Over all	**12**	**12**	**12**	**12**	**12**	**11**	**12**	**8**	**10**	**12**	**12**	**12**	**12**

Study/year	Italia 2013	Karimi 2005	Karimi 2012	Kattamis2004	Koren 2008	Kosaryan 2009	Panigrahi 2005	Rigano 2010	Singer 2005	Singer 2008	Yavarian 2004	Zamani 2009
A clearly stated aim	2	2	2	2	2	2	2	2	2	2	2	1
Inclusion of consecutive patients	2	2	2	2	2	2	1	2	2	2	2	2
Prospective collection of data	2	2	2	2	2	2	2	2	2	2	2	2
Endpoints appropriate to the aim of the study	2	2	2	2	2	2	2	2	2	2	2	2
Unbiased assessment of the study endpoint	0	0	0	0	0	0	0	0	0	0	0	0
Follow-up period appropriate to the aim of the study	2	2	2	2	2	2	1	2	2	1	2	2
Loss to follow up less than 5%	2	2	2	2	2	2	2	2	1	2	2	2
Prospective calculation of the study size	0	1	0	0	0	0	0	0	0	0	0	0
Over all	**12**	**13**	**12**	**12**	**12**	**12**	**10**	**12**	**11**	**11**	**12**	**11**
