# Supplementary material for: Efficacy and safety of hydroxyurea therapy on patients with β-thalassemia: a systematic review and meta-analysis
Source: Front Med (Lausanne). 2025 Jan 15;11:1480831. doi: 10.3389/fmed.2024.1480831 (PMC11774989; doi:10.3389/fmed.2024.1480831)
Supplement: Supplementary file 2 [file Data_Sheet_2.PDF]

Author(s): Tianmin Huang
Question: [1]
Setting:
Bibliography:

Certainty assessment							№ of patients		Effect		Certainty	Importance
№ of studies	Study design	Risk of bias	Inconsistency	Indirectness	Imprecision	Other considerations	Hydroxyurea		Relative (95% CI)	Absolute (95% CI)		
**Good response rate: Completely transfusion-free (follow-up: mean 24 months; assessed with: Completely transfusion-free)**												
1	non-randomised studies	not serious	not serious	not serious	not serious	strong association	33/119		**Rate ratio 0.28** (0.20 to 0.36)	**-- per 1000 patient(s) per years** (from -- to --)	⨁⨁⨁◯ Moderate	CRITICAL
**Adverse events (follow-up: mean 24 months; assessed with: Adverse events)**												
1	non-randomised studies	not serious	not serious	not serious	not serious	strong association	6/146		not estimable		⨁⨁⨁◯ Moderate	CRITICAL
**Response rate: the decrease of blood transfusion frequency yearly ≥50% (follow-up: mean 24 months; assessed with: blood transfusion frequency)**												
1	non-randomised studies	not serious	not serious	not serious	not serious	very strong association	90/119		**Rate ratio 0.76** (0.67 to 0.82)	**-- per 1000 patient(s) per years** (from -- to --)	⨁⨁⨁⨁ High	CRITICAL
**Response rate: The elevated hemoglobin level ≥ 1 g/dL (follow-up: mean 24 months; assessed with: hemoglobin level)**												
1	non-randomised studies	not serious	not serious	not serious	not serious	strong association	12/27		**Rate ratio 0.44** (0.28 to 0.63)	**-- per 1000 patient(s) per years** (from -- to --)	⨁⨁⨁◯ Moderate	CRITICAL

CI: confidence interval

References

1.. Ansari SH, Shamsi TS, Ashraf M, Perveen K, Farzana T, Bohray M, Erum S, Mehboob T. Efficacy of hydroxyurea in providing transfusion independence in β-thalassemia. J Pediatr Hematol Oncol. 2011 Jul;33(5):339-43. doi: 10.1097/MPH.0b013e31821b0770

Author(s): Tianmin Huang
Question: [1]
Setting:
Bibliography:

Certainty assessment							№ of patients		Effect		Certainty	Importance
**№ of studies**	**Study design**	**Risk of bias**	**Inconsistency**	**Indirectness**	**Imprecision**	**Other considerations**	**Hydroxyurea**		**Relative (95% CI)**	**Absolute (95% CI)**		
**Adverse events (follow-up: mean 28 months; assessed with: Adverse events)**												
1	non-randomised studies	not serious	not serious	not serious	not serious	very strong association	32/110		not estimable		⊕⊕⊕⊕ High	CRITICAL
**Response rate: the decrease of blood transfusion frequency yearly ≥50% (follow-up: mean 28 months; assessed with: blood transfusion frequency)**												
1	non-randomised studies	not serious	not serious	not serious	not serious	strong association	42/110		**Rate ratio 0.75** (0.67 to 0.82)	**-- per 1000 patient(s) per years** (from -- to --)	⊕⊕⊕◯ Moderate	CRITICAL

CI: confidence interval

References

1.Biswas, S., Nag, A., Ghosh, K., Ray, R., Roy, K., Bandyopadhyay, A., Bhattacharyya, M.. Genetic determinants related to pharmacological induction of foetal haemoglobin in transfusion-dependent HbE-beta thalassaemia.Ann Hematol; Feb 2019.

Author(s): Tianmin Huang
Question: [1]
Setting:
Bibliography:

Certainty assessment							№ of patients		Effect		Certainty	Importance
№ of studies	Study design	Risk of bias	Inconsistency	Indirectness	Imprecision	Other considerations	10 mg/kg/day Hydroxyurea	20 mg/kg/day Hydroxyurea	Relative (95% CI)	Absolute (95% CI)		
**Adverse events (follow-up: mean 6 months; assessed with: Adverse events)**												
1	randomised trials	serious[a]	not serious	not serious	not serious	strong association	3/32	18/29	not estimable		⨁⨁⨁⨁ High[a]	CRITICAL
**Response rate: The elevated hemoglobin level ≥ 1 g/dL (follow-up: mean 6 months; assessed with: blood transfusion frequency)**												
1	randomised trials	serious[a]	not serious	not serious	not serious	strong association	18/32	5/29	**Rate ratio 0.63** (0.47 to 0.76)	**-- per 1000 patient(s) per years** (from -- to --)	⨁⨁⨁⨁ High[a]	CRITICAL

CI: confidence interval

Explanations

a. Allocation concealment (selection bias);Blinding of participants and personnel (performance bias); Blinding of outcome assessment (detection bias)

References

1.Bohara, V. V., Ray, S., Chakrabarti, P., Ray, S. S., Nath, U. K., Chaudhuri, U.. Optimizing the dose of hydroxyurea therapy for patients with beta-thalassemia intermedia (Hb E-beta-thalassemia): a single center study from Eastern India.Hemoglobin; 2014.

Author(s): Tianmin Huang
Question: 1
Setting:
Bibliography:

Certainty assessment							№ of patients		Effect		Certainty	Importance
**№ of studies**	**Study design**	**Risk of bias**	**Inconsistency**	**Indirectness**	**Imprecision**	**Other considerations**	**Hydroxyurea**		**Relative (95% CI)**	**Absolute (95% CI)**		
**Good response rate: Completely transfusion-free (follow-up: mean 8.5 months; assessed with: Completely transfusion-free)**												
1	non-randomised studies	not serious	not serious	not serious	not serious	strong association	6/95		**Rate ratio 0.06** (0.03 to 0.13)	**-- per 1000 patient(s) per years** (from -- to --)	⨁⨁⨁◯ Moderate	CRITICAL
**Adverse events (follow-up: mean 8.5 months; assessed with: Adverse events)**												
1	non-randomised studies	not serious	not serious	not serious	not serious	very strong association	2/100		not estimable		⨁⨁⨁⨁ High	CRITICAL
**Response rate: the decrease of blood transfusion frequency yearly ≥50% (follow-up: mean 8.5 months; assessed with: blood transfusion frequency)**												
1	non-randomised studies	not serious	not serious	not serious	not serious	strong association	31/95		**Rate ratio 0.33** (0.24 to 0.43)	**-- per 1000 patient(s) per years** (from -- to --)	⨁⨁⨁◯ Moderate	CRITICAL

CI: confidence interval

References

1.Bordbar, M. R., Silavizadeh, S., Haghpanah, S., Kamfiroozi, R., Bardestani, M., Karimi, M.. Hydroxyurea Treatment in Transfusion-Dependent beta-Thalassemia Patients.Iran Red Crescent Med J; Jun 2014.

Author(s): Tianmin Huang
Question: [1]
Setting:
Bibliography:

Certainty assessment							№ of patients		Effect		Certainty	Importance
№ of studies	Study design	Risk of bias	Inconsistency	Indirectness	Imprecision	Other considerations	Hydroxyurea		Relative (95% CI)	Absolute (95% CI)		
**Response rate: the decrease of blood transfusion frequency yearly ≥50% (follow-up: mean 51 months; assessed with: blood transfusion frequency)**												
1	non-randomised studies	not serious	not serious	not serious	serious[a]	none	1/7		**Rate ratio 0.75** (0.67 to 0.82)	**-- per 1000 patient(s) per years** (from -- to --)	⨁◯◯◯ Very low[a]	CRITICAL
**Good response rate: The elevated hemoglobin level ≥ 2 g/dL (follow-up: mean 51 months; assessed with: hemoglobin level)**												
1	non-randomised studies	not serious	not serious	not serious	not serious	strong association	1/11		**Rate ratio 0.11** (0.07 to 0.19)	**-- per 1000 patient(s) per years** (from -- to --)	⨁⨁⨁◯ Moderate	CRITICAL
**Response rate: The elevated hemoglobin level ≥ 1 g/dL (follow-up: mean 51 months; assessed with: hemoglobin level)**												
1	non-randomised studies	not serious	not serious	not serious	serious[a]	strong association	3/7		**Rate ratio 0.43** (0.16 to 0.75)	**-- per 1000 patient(s) per years** (from -- to --)	⨁⨁◯◯ Low[a]	CRITICAL

CI: confidence interval

Explanations

a. Smaller sample size

References

1.. de Paula EV, Lima CS, Arruda VR, Alberto FL, Saad ST, Costa FF. Long-term hydroxyurea therapy in beta-thalassaemia patients. Eur J Haematol. 2003 Mar;70(3):151-5. doi: 10.1034/j.1600-0609.2003.00037.x.

Author(s): Tianmin Huang
Question: [1]
Setting:
Bibliography:

Certainty assessment							№ of patients		Effect		Certainty	Importance
**№ of studies**	**Study design**	**Risk of bias**	**Inconsistency**	**Indirectness**	**Imprecision**	**Other considerations**	**Hydroxyurea**		**Relative (95% CI)**	**Absolute (95% CI)**		
**Good response rate: The elevated hemoglobin level ≥ 2 g/dL (follow-up: mean 20 months; assessed with: hemoglobin level)**												
1	non-randomised studies	not serious	not serious	not serious	not serious	strong association	17/37		**Rate ratio 0.46** (0.31 to 0.62)	**-- per 1000 patient(s) per years** (from -- to --)	⊕⊕⊕◯ Moderate	CRITICAL
**Response rate: The elevated hemoglobin level ≥ 1 g/dL (follow-up: mean 20 months; assessed with: hemoglobin level)**												
1	non-randomised studies	not serious	not serious	not serious	not serious	very strong association	26/37		**Rate ratio 0.70** (0.54 to 0.83)	**-- per 1000 patient(s) per years** (from -- to --)	⊕⊕⊕⊕ High	CRITICAL

CI: confidence interval

References

1.Dixit, A., Chatterjee, T. C., Mishra, P., Choudhry, D. R., Mahapatra, M., Tyagi, S., Kabra, M., Saxena, R., Choudhry, V. P.. Hydroxyurea in thalassemia intermedia--a promising therapy.Ann Hematol; Jul 2005.

Author(s): Tianmin Huang
Question: [1]
Setting:
Bibliography:

Certainty assessment							№ of patients		Effect		Certainty	Importance
№ of studies	Study design	Risk of bias	Inconsistency	Indirectness	Imprecision	Other considerations	Hydroxyurea		Relative (95% CI)	Absolute (95% CI)		
**Good response rate: The elevated hemoglobin level ≥ 2 g/dL (follow-up: mean 6 months; assessed with: hemoglobin level)**												
1	non-randomised studies	not serious	not serious	not serious	not serious	strong association	4/16	0/0	**Rate ratio 0.25** (0.10 to 0.49)	**-- per 1000 patient(s) per years** (from -- to --)	⨁⨁⨁◯ Moderate	CRITICAL
**Response rate: The elevated hemoglobin level ≥ 1 g/dL (follow-up: mean 6 months; assessed with: hemoglobin level)**												
1	non-randomised studies	not serious	not serious	not serious	not serious	very strong association	11/16	0/0	**Rate ratio 0.69** (0.44 to 0.86)	**-- per 1000 patient(s) per years** (from -- to --)	⨁⨁⨁⨁ High	CRITICAL

CI: confidence interval

References

1.Ehsani, M. A., Hedayati-Asl, A. A., Bagheri, A., Zeinali, S., Rashidi, A.. Hydroxyurea-induced hematological response in transfusion-independent beta-thalassemia intermedia: case series and review of literature.Pediatr Hematol Oncol; Nov 2009.

Author(s): Tianmin Huang
Question: [1]
Setting:
Bibliography:

Certainty assessment							№ of patients		Effect		Certainty	Importance
№ of studies	Study design	Risk of bias	Inconsistency	Indirectness	Imprecision	Other considerations	Hydroxyurea	combined Hydroxyurea and rHuEPO	Relative (95% CI)	Absolute (95% CI)		
**Good response rate: Completely transfusion-free (follow-up: mean 12 months; assessed with: Completely transfusion-free)**												
1	randomised trials	serious[1,a]	not serious	not serious	not serious	strong association	6/40	15/40	**Rate ratio 0.15** (0.07 to 0.29)	**-- per 1000 patient(s) per years** (from -- to --)	⨁⨁⨁⨁ High[1,a]	CRITICAL
**Adverse events (follow-up: mean 12 months; assessed with: Adverse events)**												
1	randomised trials	serious[a]	not serious	not serious	not serious	very strong association	1/40	3/40	not estimable		⨁⨁⨁⨁ High[a]	CRITICAL
**Response rate: the decrease of blood transfusion frequency yearly ≥50% (follow-up: mean 12 months; assessed with: blood transfusion frequency)**												
1	randomised trials	serious[a]	not serious	not serious	not serious	strong association	25/40	29/40	**Rate ratio 0.63** (0.47 to 0.76)	**-- per 1000 patient(s) per years** (from -- to --)	⨁⨁⨁⨁ High[a]	CRITICAL

CI: confidence interval

Explanations

a. Blinding of participants and personnel (performance bias) Blinding of outcome assessment (detection bias)

References

1.Elalfy, M. S., Adly, A. A., Ismail, E. A., Elhenawy, Y. I., Elghamry, I. R.. Therapeutic superiority and safety of combined hydroxyurea with recombinant human erythropoietin over hydroxyurea in young beta-thalassemia intermedia patients.Eur J Haematol; Dec 2013.

Author(s): Tianmin Huang
Question: [1]
Setting:
Bibliography:

Certainty assessment							№ of patients		Effect		Certainty	Importance
**№ of studies**	**Study design**	**Risk of bias**	**Inconsistency**	**Indirectness**	**Imprecision**	**Other considerations**	**Hydroxyurea**		**Relative (95% CI)**	**Absolute (95% CI)**		
**Good response rate: Completely transfusion-free (follow-up: mean 50 months; assessed with: Completely transfusion-free)**												
1	non-randomised studies	not serious	not serious	not serious	not serious	strong association	11/25		**Rate ratio 0.44** (0.27 to 0.63)	**-- per 1000 patient(s) per years** (from -- to --)	⨁⨁⨁◯ Moderate	CRITICAL
**Adverse events (follow-up: mean 50 months; assessed with: Adverse events)**												
1	non-randomised studies	not serious	not serious	not serious	not serious	very strong association	2/100		not estimable		⨁⨁⨁⨁ High	CRITICAL
**Response rate: the decrease of blood transfusion frequency yearly ≥50% (follow-up: mean 50 months; assessed with: blood transfusion frequency)**												
1	non-randomised studies	not serious	not serious	not serious	not serious	strong association	23/25		**Rate ratio 0.92** (0.75 to 0.98)	**-- per 1000 patient(s) per years** (from -- to --)	⨁⨁⨁◯ Moderate	CRITICAL
**Good response rate: The elevated hemoglobin level ≥ 2 g/dL (follow-up: mean 50 months; assessed with: hemoglobin level)**												
1	non-randomised studies	not serious	not serious	not serious	not serious	strong association	22/75		**Rate ratio 0.29** (0.20 to 0.40)	**-- per 1000 patient(s) per years** (from -- to --)	⨁⨁⨁◯ Moderate	CRITICAL
**Response rate: The elevated hemoglobin level ≥ 1 g/dL (follow-up: mean 50 months; assessed with: hemoglobin level)**												
1	non-randomised studies	not serious	not serious	not serious	not serious	very strong association	56/75		**Rate ratio 0.75** (0.64 to 0.83)	**-- per 1000 patient(s) per years** (from -- to --)	⨁⨁⨁⨁ High	CRITICAL

CI: confidence interval

References

1.El-Beshlawy, A., El-Ghamrawy, M., MA, E.,L-Ela, Said, F., Adolf, S., Abdel-Razek, A. R., Magdy, R. I., Abdel-Salam, A.. Response to hydroxycarbamide in pediatric beta-thalassemia intermedia: 8 years' follow-up in Egypt.Ann Hematol; Dec 2014.

Author(s): Tianmin Huang
Question: [1]
Setting:
Bibliography:

Certainty assessment							№ of patients		Effect		Certainty	Importance
**№ of studies**	**Study design**	**Risk of bias**	**Inconsistency**	**Indirectness**	**Imprecision**	**Other considerations**	**Hydroxyurea**		**Relative (95% CI)**	**Absolute (95% CI)**		
**Adverse events (follow-up: mean 5 months; assessed with: Adverse events)**												
1	non-randomised studies	not serious	not serious	not serious	not serious	strong association	3/13	0/0	not estimable		⨁⨁⨁◯ Moderate	CRITICAL
**Good response rate: The elevated hemoglobin level ≥ 2 g/dL (follow-up: mean 5 months; assessed with: hemoglobin level)**												
1	non-randomised studies	not serious	not serious	not serious	not serious	none	0/13	0/0	**Rate ratio 0.00** (0.00 to 0.23)	**-- per 1000 patient(s) per years** (from -- to --)	⨁⨁◯◯ Low	CRITICAL
**Response rate: The elevated hemoglobin level ≥ 1 g/dL (follow-up: mean 5 months; assessed with: hemoglobin level)**												
1	non-randomised studies	not serious	not serious	not serious	not serious	strong association	3/13	0/0	**Rate ratio 0.23** (0.08 to 0.50)	**-- per 1000 patient(s) per years** (from -- to --)	⨁⨁⨁◯ Moderate	CRITICAL

CI: confidence interval

References

1.. Fucharoen S, Siritanaratkul N, Winichagoon P, Chowthaworn J, Siriboon W, Muangsup W, Chaicharoen S, Poolsup N, Chindavijak B, Pootrakul P, Piankijagum A, Schechter AN, Rodgers GP. Hydroxyurea increases hemoglobin F levels and improves the effectiveness of erythropoiesis in beta-thalassemia/hemoglobin E disease. Blood. 1996 Feb 1;87(3):887-92.

Author(s): Tianmin Huang
Question: [1]
Setting:
Bibliography:

Certainty assessment							№ of patients		Effect		Certainty	Importance
№ of studies	Study design	Risk of bias	Inconsistency	Indirectness	Imprecision	Other considerations	Hydroxyurea		Relative (95% CI)	Absolute (95% CI)		
**Good response rate: Completely transfusion-free (follow-up: mean 24 months; assessed with: Completely transfusion-free)**												
1	non-randomised studies	not serious	not serious	not serious	not serious	strong association	17/91		**Rate ratio 0.19** (0.12 to 0.28)	**-- per 1000 patient(s) per years** (from -- to --)	⨁⨁⨁◯ Moderate	CRITICAL
**Response rate: the decrease of blood transfusion frequency yearly ≥50% (follow-up: mean 24 months; assessed with: blood transfusion frequency)**												
1	non-randomised studies	not serious	not serious	not serious	not serious	strong association	38/91		**Rate ratio 0.42** (0.32 to 0.52)	**-- per 1000 patient(s) per years** (from -- to --)	⨁⨁⨁◯ Moderate	CRITICAL

CI: confidence interval

References

1.. Azamsadat Hashemi, Mahboobeh Abrishamkar, Ali Reza Jenabzade, Ziae Eslami. Hydroxyurea Can Reduce or Eliminate Transfusion Requirements in Children with Major and Intermediate Thalassemia. IJBC 2009; 4: 147-150.

Author(s): Tianmin Huang
Question: [1]
Setting:
Bibliography:

Certainty assessment							№ of patients		Effect		Certainty	Importance
№ of studies	Study design	Risk of bias	Inconsistency	Indirectness	Imprecision	Other considerations	Hydroxyurea		Relative (95% CI)	Absolute (95% CI)		
**Good response rate: Completely transfusion-free (follow-up: mean 12 months; assessed with: Completely transfusion-free)**												
1	non-randomised studies	not serious	not serious	not serious	not serious	very strong association	10/12		**Rate ratio 0.83** (0.55 to 0.95)	**-- per 1000 patient(s) per years** (from -- to --)	⨁⨁⨁⨁ High	CRITICAL
**adverse event (follow-up: mean 12 months; assessed with: adverse event)**												
1	non-randomised studies	not serious	not serious	not serious	not serious	strong association	4/14 (28.6%)		not estimable		⨁⨁⨁◯ Moderate	CRITICAL

CI: confidence interval

References

1.. Huang L, Yao HX. Curative Effects of Hydroxyurea on the Patients with β-thalassaemia Intermadia[J]. Journal of Experimental Hematology, 2016, 24(3): 806-809.

Author(s): Tianmin Huang
Question: [1]
Setting:
Bibliography:

Certainty assessment							№ of patients		Effect		Certainty	Importance
№ of studies	Study design	Risk of bias	Inconsistency	Indirectness	Imprecision	Other considerations	Hydroxyurea		Relative (95% CI)	Absolute (95% CI)		
**Good response rate: Completely transfusion-free (follow-up: mean 9 months; assessed with: Completely transfusion-free)**												
1	non-randomised studies	not serious	not serious	not serious	not serious	strong association	25/70		**Rate ratio 0.36** (0.26 to 0.47)	**-- per 1000 patient(s) per years** (from -- to --)	⨁⨁⨁◯ Moderate	CRITICAL
**Response rate: the decrease of blood transfusion frequency yearly ≥50% (follow-up: mean 9 months; assessed with: blood transfusion frequency)**												
1	non-randomised studies	not serious	not serious	not serious	not serious	very strong association	48/70		**Rate ratio 0.75** (0.67 to 0.82)	**-- per 1000 patient(s) per years** (from -- to --)	⨁⨁⨁⨁ High	CRITICAL

CI: confidence interval

References

1.Iqbal, A., Ansari, S. H., Parveen, S., Khan, I. A., Siddiqui, A. J., Musharraf, S. G.. Hydroxyurea Treated beta-Thalassemia Children Demonstrate a Shift in Metabolism Towards Healthy Pattern.Sci Rep; Oct 11 2018.

Author(s): Tianmin Huang
Question: [1]
Setting:
Bibliography:

Certainty assessment							№ of patients		Effect		Certainty	Importance
№ of studies	Study design	Risk of bias	Inconsistency	Indirectness	Imprecision	Other considerations	Hydroxyurea		Relative (95% CI)	Absolute (95% CI)		
**Adverse events (follow-up: mean 20 months; assessed with: Adverse events)**												
1	non-randomised studies	not serious	not serious	not serious	not serious	strong association	12/79		not estimable		⨁⨁⨁◯ Moderate	CRITICAL
**Response rate: the decrease of blood transfusion frequency yearly ≥50% (follow-up: mean 20 months; assessed with: blood transfusion frequency)**												
1	non-randomised studies	not serious	not serious	not serious	not serious	strong association	13/41	0/0	**Rate ratio 0.32** (0.20 to 0.47)	**-- per 1000 patient(s) per years** (from -- to --)	⨁⨁⨁◯ Moderate	CRITICAL

CI: confidence interval

References

1.Italia, K. Y., Jijina, F. J., Merchant, R., Panjwani, S., Nadkarni, A. H., Sawant, P. M., Nair, S. B., Ghosh, K., Colah, R. B.. Response to hydroxyurea in beta thalassemia major and intermedia: experience in western India.Clin Chim Acta; Sep 2009.

Author(s): Tianmin Huang
Question: [1]
Setting:
Bibliography:

Certainty assessment							№ of patients		Effect		Certainty	Importance
№ of studies	Study design	Risk of bias	Inconsistency	Indirectness	Imprecision	Other considerations	Hydroxyurea		Relative (95% CI)	Absolute (95% CI)		
**Good response rate: Completely transfusion-free (follow-up: mean 20 months; assessed with: Completely transfusion-free)**												
1	non-randomised studies	not serious	not serious	not serious	not serious	strong association	4/11		**Rate ratio 0.36** (0.15 to 0.65)	**-- per 1000 patient(s) per years** (from -- to --)	⨁⨁⨁◯ Moderate	CRITICAL

CI: confidence interval

References

1.Italia, K. Y., Jijina, F. F., Merchant, R., Panjwani, S., Nadkarni, A. H., Sawant, P. M., Nair, S. B., Ghosh, K., Colah, R. B.. Effect of hydroxyurea on the transfusion requirements in patients with severe HbE-beta-thalassaemia: a genotypic and phenotypic study.J Clin Pathol; Feb 2010.

Author(s): Tianmin Huang
Question: [1]
Setting:
Bibliography:

Certainty assessment							№ of patients		Effect		Certainty	Importance
**№ of studies**	**Study design**	**Risk of bias**	**Inconsistency**	**Indirectness**	**Imprecision**	**Other considerations**	**Hydroxyurea**		**Relative (95% CI)**	**Absolute (95% CI)**		
**Good response rate: Completely transfusion-free (follow-up: mean 24 months; assessed with: Completely transfusion-free)**												
1	non-randomised studies	not serious	not serious	not serious	not serious	strong association	2/11		**Rate ratio 0.18** (0.05 to 0.48)	**-- per 1000 patient(s) per years** (from -- to --)	⨁⨁⨁◯ Moderate	CRITICAL
**Adverse events (follow-up: mean 24 months; assessed with: Adverse events)**												
1	non-randomised studies	not serious	not serious	not serious	not serious	strong association	2/16		not estimable		⨁⨁⨁◯ Moderate	CRITICAL
**Response rate: the decrease of blood transfusion frequency yearly ≥50% (follow-up: mean 24 months; assessed with: blood transfusion frequency)**												
1	non-randomised studies	not serious	not serious	not serious	not serious	very strong association	7/11		**Rate ratio 0.64** (0.32 to 0.85)	**-- per 1000 patient(s) per years** (from -- to --)	⨁⨁⨁⨁ High	CRITICAL

CI: confidence interval

References

1.Italia, K., Jijina, F., Merchant, R., Swaminathan, S., Nadkarni, A., Gupta, M., Ghosh, K., Colah, R.. Comparison of in-vitro and in-vivo response to fetal hemoglobin production and gamma-mRNA expression by hydroxyurea in Hemoglobinopathies.Indian J Hum Genet; Apr 2013.

Author(s): Tianmin Huang
Question: [1]
Setting:
Bibliography:

Certainty assessment							№ of patients		Effect		Certainty	Importance
№ of studies	Study design	Risk of bias	Inconsistency	Indirectness	Imprecision	Other considerations	Hydroxyurea		Relative (95% CI)	Absolute (95% CI)		
**Good response rate: Completely transfusion-free (follow-up: mean 72 months; assessed with: Completely transfusion-free)**												
1	non-randomised studies	not serious	not serious	not serious	not serious	strong association	83/120		**Rate ratio 0.69** (0.60 to 0.70)	**-- per 1000 patient(s) per years** (from -- to --)	⨁⨁⨁◯ Moderate	CRITICAL
**Response rate: the decrease of blood transfusion frequency yearly ≥50% (follow-up: mean 72 months; assessed with: blood transfusion frequency)**												
1	non-randomised studies	not serious	not serious	not serious	not serious	very strong association	106/120		**Rate ratio 0.88** (0.81 to 0.93)	**-- per 1000 patient(s) per years** (from -- to --)	⨁⨁⨁⨁ High	CRITICAL

CI: confidence interval

References

1.. Karimi M, Darzi H, Yavarian M. Hematologic and clinical responses of thalassemia intermedia patients to hydroxyurea during 6 years of therapy in Iran. J Pediatr Hematol Oncol. 2005 Jul;27(7):380-5. doi: 10.1097/01.mph.0000174386.13109.28.

Author(s): Tianmin Huang
Question: [1]
Setting:
Bibliography:

Certainty assessment							№ of patients		Effect		Certainty	Importance
№ of studies	Study design	Risk of bias	Inconsistency	Indirectness	Imprecision	Other considerations	Hydroxyurea		Relative (95% CI)	Absolute (95% CI)		
**Good response rate: Completely transfusion-free (follow-up: mean 156 months; assessed with: Completely transfusion-free)**												
1	non-randomised studies	not serious	not serious	not serious	not serious	strong association	51/126		**Rate ratio 0.40** (0.32 to 0.49)	**-- per 1000 patient(s) per years** (from -- to --)	⨁⨁⨁◯ Moderate	CRITICAL
**Adverse events (follow-up: mean 156 months; assessed with: Adverse events)**												
1	non-randomised studies	not serious	not serious	not serious	not serious	very strong association	57/232		not estimable		⨁⨁⨁⨁ High	CRITICAL
**Response rate: the decrease of blood transfusion frequency yearly ≥50% (follow-up: mean 156 months; assessed with: blood transfusion frequency)**												
1	non-randomised studies	not serious	not serious	not serious	not serious	very strong association	95/126		**Rate ratio 0.75** (0.67 to 0.82)	**-- per 1000 patient(s) per years** (from -- to --)	⨁⨁⨁⨁ High	CRITICAL
**Good response rate: The elevated hemoglobin level ≥ 2 g/dL (follow-up: mean 156 months; assessed with: hemoglobin level)**												
1	non-randomised studies	not serious	not serious	not serious	not serious	strong association	12/106		**Rate ratio 0.11** (0.07 to 0.19)	**-- per 1000 patient(s) per years** (from -- to --)	⨁⨁⨁◯ Moderate	CRITICAL
**Response rate: The elevated hemoglobin level ≥ 1 g/dL (follow-up: mean 156 months; assessed with: hemoglobin level)**												
1	non-randomised studies	not serious	not serious	not serious	not serious	very strong association	56/106		**Rate ratio 0.53** (0.43 to 0.62)	**-- per 1000 patient(s) per years** (from -- to --)	⨁⨁⨁⨁ High	CRITICAL

CI: confidence interval

References

1.Karimi, M., Haghpanah, S., Farhadi, A., Yavarian, M.. Genotype-phenotype relationship of patients with beta-thalassemia taking hydroxyurea: a 13-year experience in Iran.Int J Hematol; Jan 2012.

Author(s): Tianmin Huang
Question: [1]
Setting:
Bibliography:

Certainty assessment							№ of patients		Effect		Certainty	Importance
**№ of studies**	**Study design**	**Risk of bias**	**Inconsistency**	**Indirectness**	**Imprecision**	**Other considerations**	**Hydroxyurea**		**Relative (95% CI)**	**Absolute (95% CI)**		
**Good response rate: Completely transfusion-free (follow-up: mean 24 months; assessed with: Completely transfusion-free)**												
1	non-randomised studies	not serious	not serious	not serious	not serious	none	0/8		**Rate ratio 0.00** (0.00 to 0.32)	**-- per 1000 patient(s) per years** (from -- to --)	⨁⨁◯◯ Low	CRITICAL
**Adverse events (follow-up: mean 24 months; assessed with: Adverse events)**												
1	non-randomised studies	not serious	not serious	not serious	not serious	strong association	2/8		not estimable		⨁⨁⨁◯ Moderate	CRITICAL
**Response rate: the decrease of blood transfusion frequency yearly ≥50% (follow-up: mean 24 months; assessed with: blood transfusion frequency)**												
1	non-randomised studies	not serious	not serious	not serious	not serious	strong association	2/8		**Rate ratio 0.25** (0.07 to 0.59)	**-- per 1000 patient(s) per years** (from -- to --)	⨁⨁⨁◯ Moderate	CRITICAL

CI: confidence interval

References

1.Kattamis, A., Lagona, E., Orfanou, I., Psichou, F., Ladis, V., Kanavakis, E., Metaxotou-Mavrommati, A., Kattamis, C.. Clinical response and adverse events in young patients with sickle cell disease treated with hydroxyurea.Pediatr Hematol Oncol; Jun 2004.

Author(s): Tianmin Huang
Question: [1]
Setting:
Bibliography:

Certainty assessment							№ of patients		Effect		Certainty	Importance
№ of studies	Study design	Risk of bias	Inconsistency	Indirectness	Imprecision	Other considerations	Hydroxyurea		Relative (95% CI)	Absolute (95% CI)		
**Good response rate: Completely transfusion-free (follow-up: mean 48 months; assessed with: Completely transfusion-free)**												
1	non-randomised studies	not serious	not serious	not serious	not serious	very strong association	14/16		**Rate ratio 0.88** (0.64 to 0.97)	**-- per 1000 patient(s) per years** (from -- to --)	⨁⨁⨁⨁ High	CRITICAL
**Adverse events (follow-up: mean 48 months; assessed with: Adverse events)**												
1	non-randomised studies	not serious	not serious	not serious	not serious	strong association	3/18		not estimable		⨁⨁⨁◯ Moderate	CRITICAL
**Response rate: the decrease of blood transfusion frequency yearly ≥50% (follow-up: mean 48 months; assessed with: blood transfusion frequency)**												
1	non-randomised studies	not serious	not serious	not serious	not serious	very strong association	14/16		**Rate ratio 0.88** (0.64 to 0.97)	**-- per 1000 patient(s) per years** (from -- to --)	⨁⨁⨁⨁ High	CRITICAL

CI: confidence interval

References

1.Koren, A., Levin, C., Dgany, O., Kransnov, T., Elhasid, R., Zalman, L., Palmor, H., Tamary, H.. Response to hydroxyurea therapy in beta-thalassemia.Am J Hematol; May 2008.

Author(s): Tianmin Huang
Question: Efficacy and Safety of Hydroxyurea Therapy on Patients With β-Thalassemia[1]
Setting:
Bibliography:

Certainty assessment							№ of patients		Effect		Certainty	Importance
№ of studies	Study design	Risk of bias	Inconsistency	Indirectness	Imprecision	Other considerations	Hydroxyurea		Relative (95% CI)	Absolute (95% CI)		
**Good response rate: Completely transfusion-free (follow-up: mean 89.5 months; assessed with: Completely transfusion-free)**												
1	non-randomised studies	not serious	not serious	not serious	not serious	strong association	111/248		**Rate ratio 0.45** (0.39 to 0.51)	**-- per 1000 patient(s) per years** (from -- to --)	⨁⨁⨁◯ Moderate	CRITICAL
**Adverse events (follow-up: mean 89.5 months; assessed with: Adverse events)**												
1	non-randomised studies	not serious	not serious	not serious	not serious	strong association	18/248		not estimable		⨁⨁⨁◯ Moderate	CRITICAL

CI: confidence interval

References

1.Kosaryan, M., Zafari, M., Alipur, A., Hedayatizadeh-Omran, A.. The effect and side effect of hydroxyurea therapy on patients with beta-thalassemia: a systematic review to December 2012.Hemoglobin; 2014.

Author(s): Tianmin Huang
Question: Efficacy and Safety of Hydroxyurea Therapy on Patients With β-Thalassemia[1]
Setting:
Bibliography:

Certainty assessment							№ of patients		Effect		Certainty	Importance
**№ of studies**	**Study design**	**Risk of bias**	**Inconsistency**	**Indirectness**	**Imprecision**	**Other considerations**	**Hydroxyurea**		**Relative (95% CI)**	**Absolute (95% CI)**		
**Response rate: the in crease of hemoglobin level ≥1g/dl (follow-up: mean 12 months; assessed with: Hb)**												
1	non-randomised studies	not serious	not serious	not serious	not serious	strong association	8/15		**Rate ratio 0.53** (0.30 to 0.75)	**-- per 1000 patient(s) per years** (from -- to --)	⨁⨁⨁◯ Moderate	CRITICAL

CI: confidence interval

References

1.Panigrahi, I., Dixit, A., Arora, S., Kabra, M., Mahapatra, M., Choudhry, V. P., Saxena, R.. Do alpha deletions influence hydroxyurea response in thalassemia intermedia?.Hematology; Feb 2005.

Author(s): Tianmin Huang
Question: Efficacy and Safety of Hydroxyurea Therapy on Patients With β-Thalassemia[1]
Setting: [1]
Bibliography:

Certainty assessment							№ of patients		Effect		Certainty	Importance
№ of studies	Study design	Risk of bias	Inconsistency	Indirectness	Imprecision	Other considerations	Hydroxyurea		Relative (95% CI)	Absolute (95% CI)		
**Response rate: the in crease of hemoglobin level ≥1g/dl (follow-up: mean 80 months; assessed with: Hb)**												
1	non-randomised studies	not serious	not serious	not serious	not serious	very strong association	17/24		**Rate ratio 0.71** (0.51 to 0.85)	**-- per 1000 patient(s) per years** (from -- to --)	⨁⨁⨁⨁ High	CRITICAL

CI: confidence interval

References

1.Rigano, P., Pecoraro, A., Calzolari, R., Troia, A., Acuto, S., Renda, D., Pantalone, G. R., Maggio, A., Di Marzo, R.. Desensitization to hydroxycarbamide following long-term treatment of thalassaemia intermedia as observed in vivo and in primary erythroid cultures from treated patients.Br J Haematol; Dec 2010.

Author(s): Tianmin Huang
Question: [1]
Setting:
Bibliography:

Certainty assessment							№ of patients		Effect		Certainty	Importance
№ of studies	Study design	Risk of bias	Inconsistency	Indirectness	Imprecision	Other considerations	Hydroxyurea		Relative (95% CI)	Absolute (95% CI)		
**Good response rate: Completely transfusion-free (follow-up: mean 36 months; assessed with: Completely transfusion-free)**												
1	non-randomised studies	not serious	not serious	not serious	not serious	strong association	12/27		**Rate ratio 0.44** (0.25 to 0.63)	**-- per 1000 patient(s) per years** (from -- to --)	⨁⨁⨁◯ Moderate	CRITICAL

CI: confidence interval

References

1.Singer, S. T., Kuypers, F. A., Olivieri, N. F., Weatherall, D. J., Mignacca, R., Coates, T. D., Davies, S., Sweeters, N., Vichinsky, E. P., Group, E.,beta,Thalassaemia,Study. Fetal haemoglobin augmentation in E/beta(0) thalassaemia: clinical and haematological outcome.Br J Haematol; Nov 2005.

Author(s): Tianmin Huang
Question: Efficacy and Safety of Hydroxyurea Therapy on Patients With β-Thalassemia[1]
Setting:
Bibliography: [1]

Certainty assessment							№ of patients		Effect		Certainty	Importance
№ of studies	Study design	Risk of bias	Inconsistency	Indirectness	Imprecision	Other considerations	Hydroxyurea		Relative (95% CI)	Absolute (95% CI)		
**Response rate: the in crease of hemoglobin level ≥1g/dl (follow-up: mean 10.2 months; assessed with: Hb)**												
1	non-randomised studies	not serious	not serious	not serious	not serious	strong association	8/15		**Rate ratio 0.53** (0.30 to 0.75)	**-- per 100 patient(s) per months** (from -- to --)	⨁⨁⨁◯ Moderate	CRITICAL

CI: confidence interval

References

1.Singer, S. T., Vichinsky, E. P., Larkin, S., Olivieri, N., Sweeters, N., Kuypers, F. A., Group, E.,beta,Thalassemia,Study. Hydroxycarbamide-induced changes in E/beta thalassemia red blood cells.Am J Hematol; Nov 2008.

Author(s): Tianmin Huang
Question: [1]
Setting:
Bibliography:

Certainty assessment							№ of patients		Effect		Certainty	Importance
№ of studies	Study design	Risk of bias	Inconsistency	Indirectness	Imprecision	Other considerations	Hydroxyurea		Relative (95% CI)	Absolute (95% CI)		
**Good response rate: Completely transfusion-free (follow-up: mean 60 months; assessed with: Completely transfusion-free)**												
1	non-randomised studies	not serious	not serious	not serious	not serious	very strong association	81/133		**Rate ratio 0.61** (0.52 to 0.69)	**-- per 1000 patient(s) per years** (from -- to --)	⨁⨁⨁⨁ High	CRITICAL
**Response rate: the decrease of blood transfusion frequency yearly ≥50% (follow-up: mean 60 months; assessed with: blood transfusion frequency)**												
1	non-randomised studies	not serious	not serious	not serious	not serious	very strong association	112/133		**Rate ratio 0.84** (0.77 to 0.89)	**-- per 1000 patient(s) per years** (from -- to --)	⨁⨁⨁⨁ High	CRITICAL

CI: confidence interval

References

1.. Yavarian M, Karimi M, Bakker E, Harteveld CL, Giordano PC. Response to hydroxyurea treatment in Iranian transfusion-dependent beta-thalassemia patients. Haematologica. 2004 Oct;89(10):1172-8. PMID: 15477200.

Author(s): Tianmin Huang
Question: [1]
Setting:
Bibliography:

Certainty assessment							№ of patients		Effect		Certainty	Importance
**№ of studies**	**Study design**	**Risk of bias**	**Inconsistency**	**Indirectness**	**Imprecision**	**Other considerations**	**Hydroxyurea**		**Relative (95% CI)**	**Absolute (95% CI)**		
**Good response rate: Completely transfusion-free (follow-up: mean 60 months; assessed with: Completely transfusion-free)**												
1	non-randomised studies	not serious	not serious	not serious	not serious	strong association	12/49	0/0	**Rate ratio 0.40** (0.32 to 0.49)	**-- per 1000 patient(s) per years** (from -- to --)	⨁⨁⨁◯ Moderate	CRITICAL
**Adverse events (follow-up: mean 60 months; assessed with: Adverse events)**												
1	non-randomised studies	not serious	not serious	not serious	not serious	strong association	9/49	0/0	not estimable		⨁⨁⨁◯ Moderate	CRITICAL
**Response rate: the decrease of blood transfusion frequency yearly ≥50% (follow-up: mean 60 months; assessed with: blood transfusion frequency)**												
1	non-randomised studies	not serious	not serious	not serious	not serious	very strong association	44/49	0/0	**Rate ratio 0.75** (0.67 to 0.82)	**-- per 1000 patient(s) per years** (from -- to --)	⨁⨁⨁⨁ High	CRITICAL

CI: confidence interval

References

1.. Zamani F, Shakeri R, Eslami SM, Razavi SM, Basi A. Hydroxyurea therapy in 49 patients with major beta-thalassemia. Arch Iran Med. 2009 May;12(3):295-7. PMID: 19400608.
