# Supplementary material for: Efficacy and safety of hydroxyurea therapy on patients with β-thalassemia: a systematic review and meta-analysis
Source: Front Med (Lausanne). 2025 Jan 15;11:1480831. doi: 10.3389/fmed.2024.1480831 (PMC11774989; doi:10.3389/fmed.2024.1480831)
Supplement: Supplementary file 3 [file Data_Sheet_3.PDF]

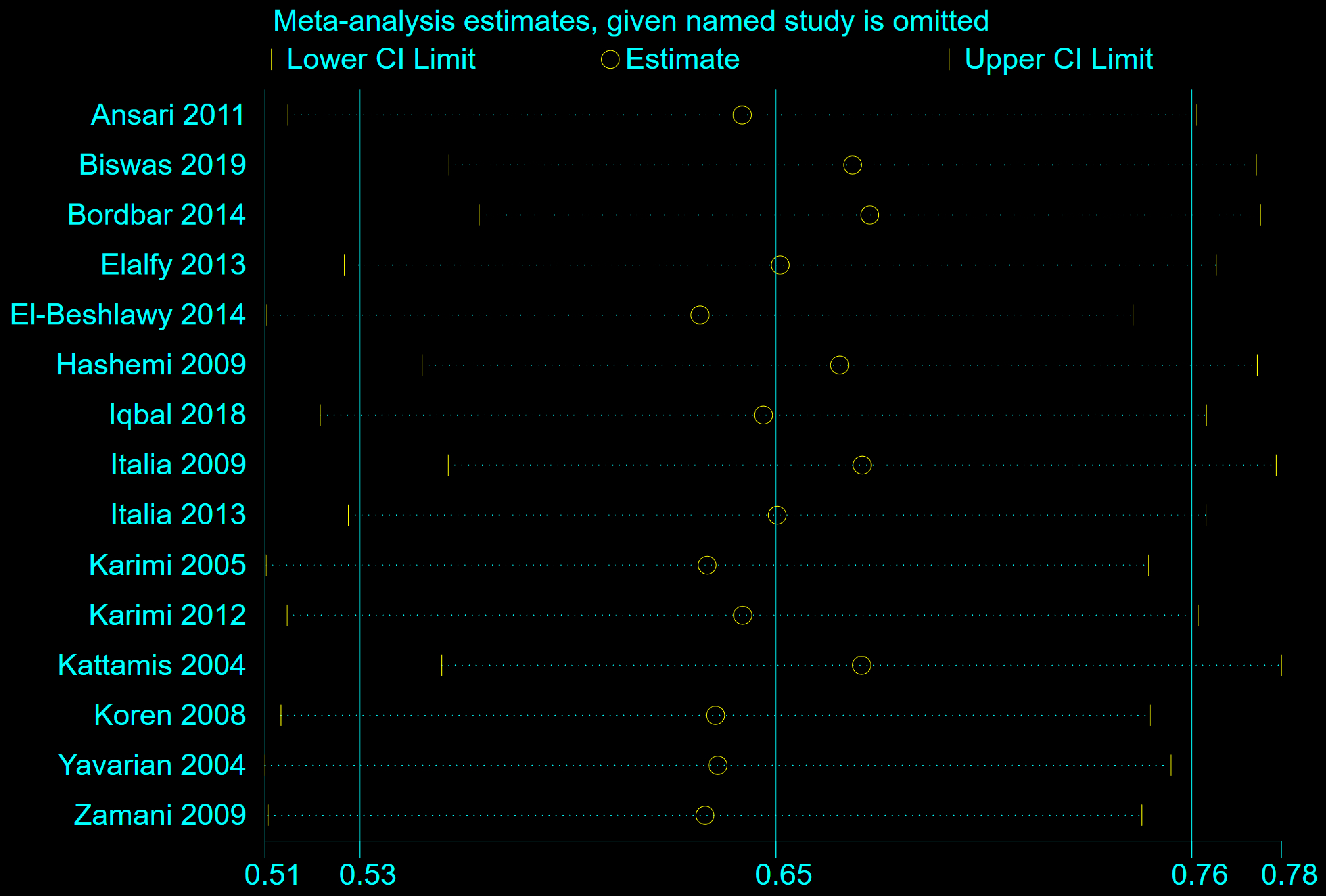

A. The response rate of HU in transfusion-dependentβ-thalassemia

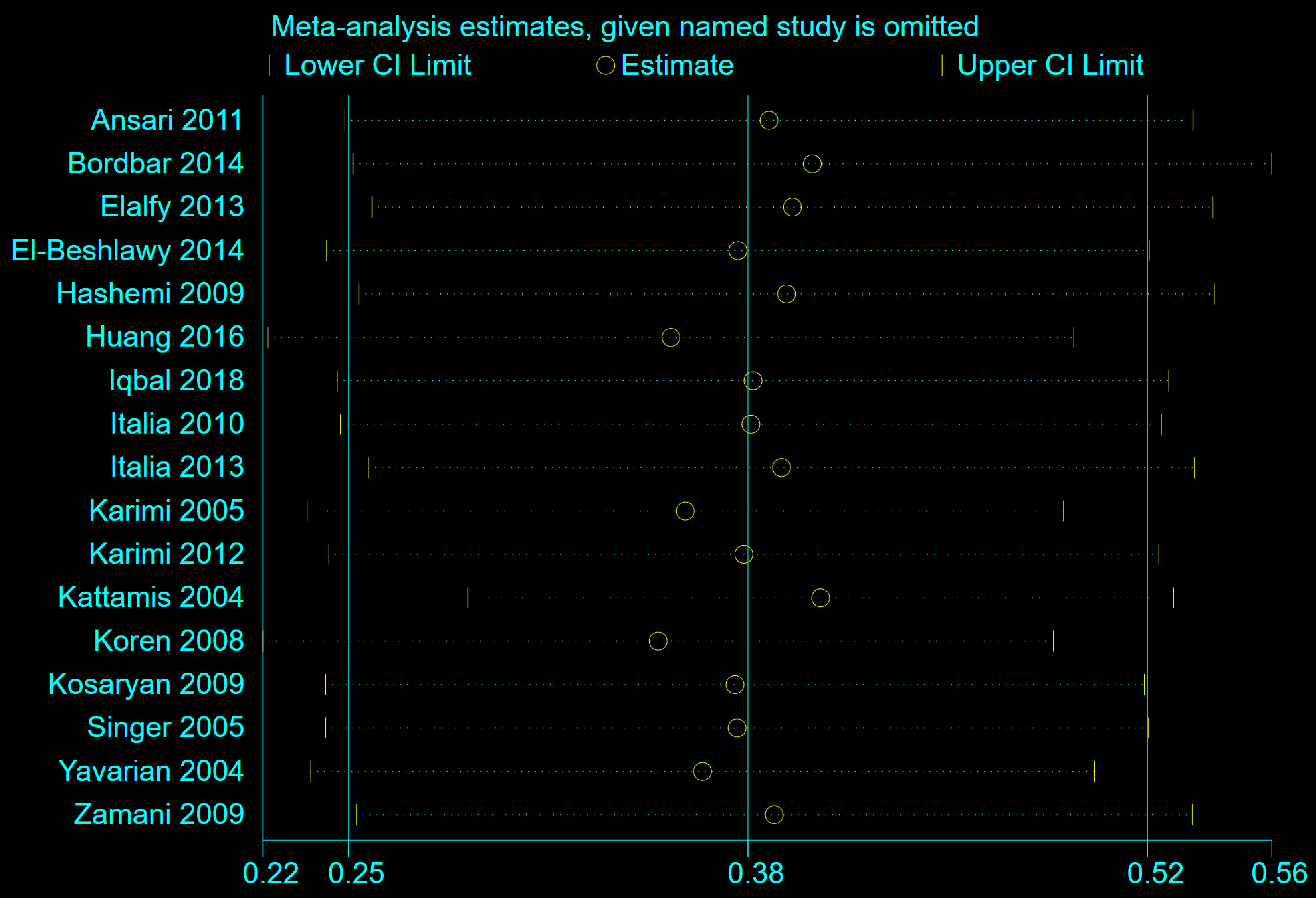

B. The good response rate of HU in transfusion-dependentβ-thalassemia

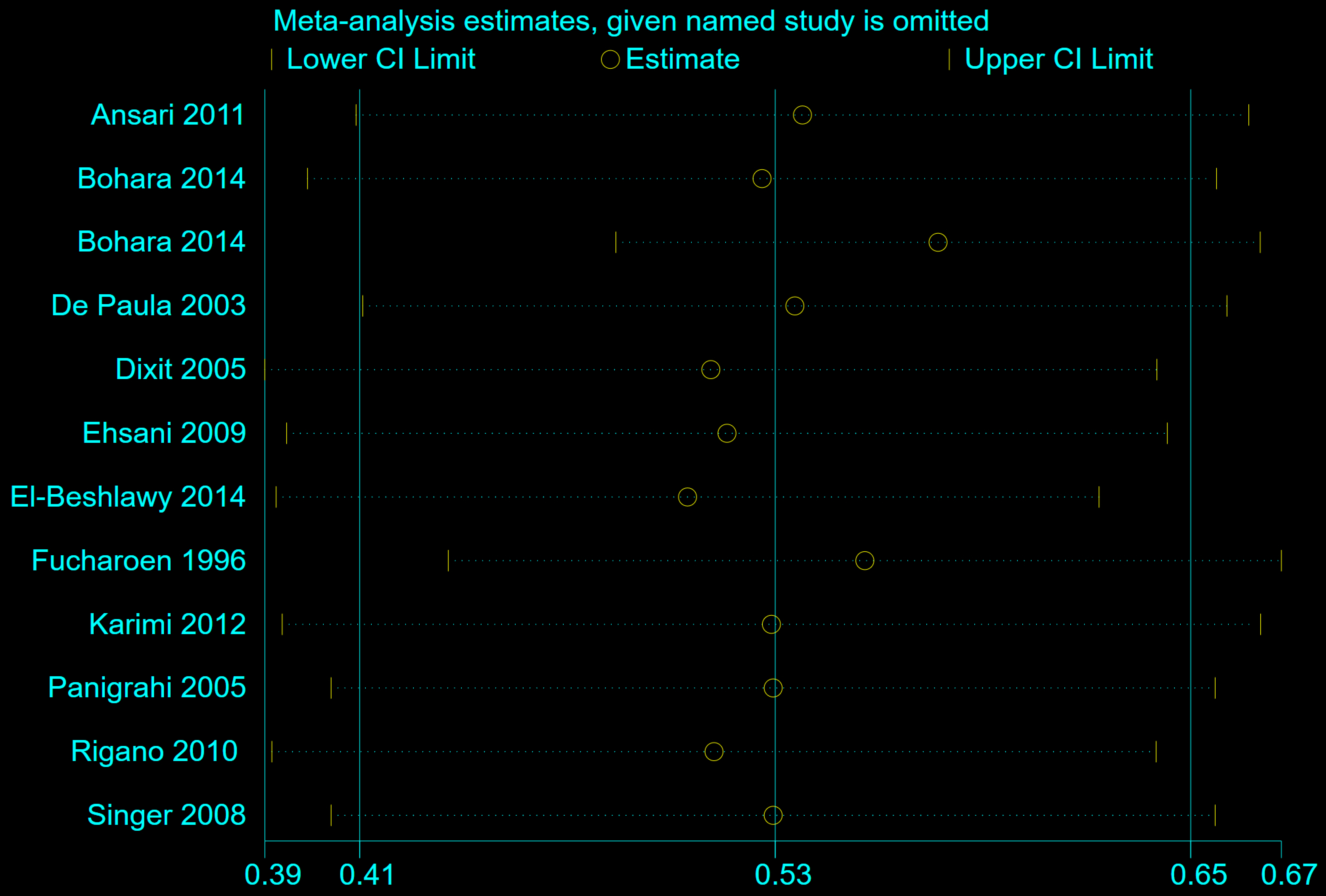

C. The response rate of HU in intransfusion-independentβ-thalassemia

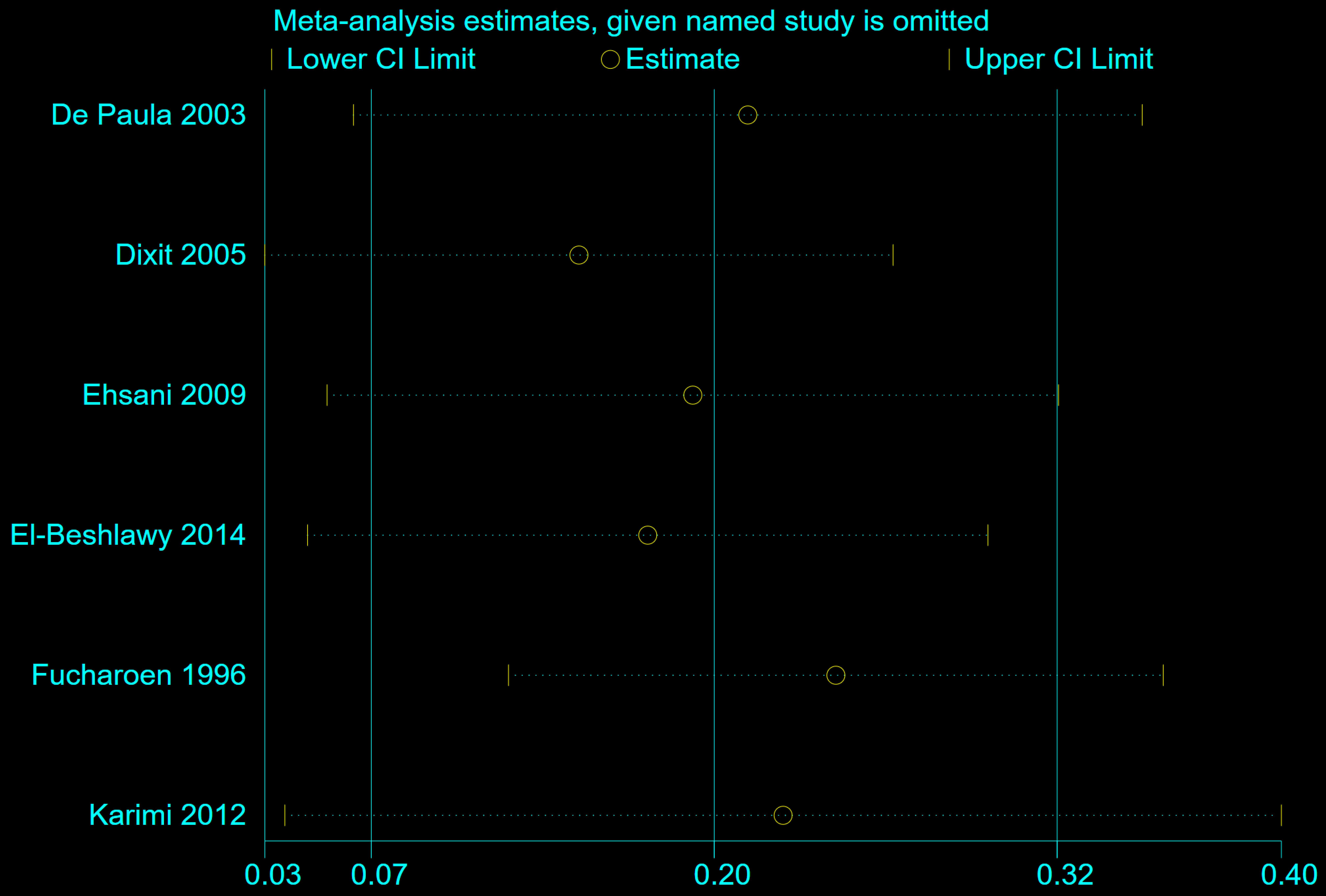

D. The good response rate of HU in intransfusion-independentβ-thalassemia
